# Supplementary material for: Quality assessment of clinical practice guidelines in Kenya using the AGREE II tool: a methodological review
Source: BMJ Open. 2023 Jul 10;13(7):e074510. doi: 10.1136/bmjopen-2023-074510 (PMC10335456; doi:10.1136/bmjopen-2023-074510)
Supplement: Supplementary data [file bmjopen-2023-074510supp001.pdf]

**Supplementary file 1: AGREE II Instrument domains and items**

| Domain | Item no. | Item |
|---|---|---|
| 1. Scope and purpose | 1. | The guideline's overall objective(s) is (are) specifically described. |
| | 2. | The health question(s) covered by the guideline is (are) specifically described. |
| | 3. | The population (patients, public, etc.) the guideline is meant to apply is specifically described. |
| 2. Stakeholder involvement | 4. | The guideline development group includes individuals from all relevant professional groups. |
| | 5. | The views and preferences of the target population (patients, the public, etc.) have been sought. |
| | 6. | The target users of the guideline are clearly defined. |
| 3. Rigour of development | 7. | Systematic methods were used to search for evidence. |
| | 8. | The criteria for selecting the evidence are clearly described. |
| | 9. | The strengths and limitations of the body of evidence are clearly described. |
| | 10. | The methods for formulating the recommendations are clearly described. |
| | 11. | The health benefits, side effects, and risks have been considered in formulating the recommendations. |
| | 12. | There is an explicit link between the recommendations and the supporting evidence. |
| | 13. | Experts have externally reviewed the guideline before its publication. |
| | 14. | A procedure for updating the guideline is provided. |
| 4. Clarity of Presentation | 15. | The recommendations are specific and unambiguous. |
| | 16. | The different options for management of the condition or health issue are clearly presented. |
| | 17. | Key recommendations are easily identifiable. |
| 5. Applicability | 18. | The guideline describes the facilitators and barriers to its application. |
| | 19. | The guideline provides advice and/or tools on how the recommendations can be implemented. |

| | | |
|---|---|---|
| | 20. | The potential resource implications of applying the recommendations have been considered. |
| | 21. | The guideline presents monitoring and/or auditing criteria. |
| 6. Editorial Independence | 22. | The views of the funding body have not influenced the guideline's content. |
| | 23. | Competing interests of guideline development group members have been recorded and addressed |
| Overall guideline Assessment | | Rate the overall quality of this guideline. |
| | | I would recommend this guideline for use. (Yes, yes with modifications, no). |
