# Supplementary material for: Quality assessment of clinical practice guidelines in Kenya using the AGREE II tool: a methodological review
Source: BMJ Open. 2023 Jul 10;13(7):e074510. doi: 10.1136/bmjopen-2023-074510 (PMC10335456; doi:10.1136/bmjopen-2023-074510)
Supplement: Supplementary data [file bmjopen-2023-074510supp002.pdf]

Supplementary file 2: Websites, databases and experts contacted in clinical practice guidelines search

	**Disease**	**Databases**	**Contact persons/Experts**	**Type of search**
1.	HIV	Ministry of health<br>National aids control council (NCC)<br>Division of National AIDS and STI Control Programme (NASCOP)	Ministry of health officials<br>Respective heads	Hand searching
2.	T.B.	Ministry of health<br>Centre for Health Solutions (CHS) Kenya<br>Kenya Association for the Prevention of T.B. and Lung Diseases<br>Division of National T.B., Leprosy and Lung disease programme	Ministry of health officials respective heads	Hand searching
3.	Malaria	Ministry of health<br>The Division of National Malaria Programme (DNMP)	Ministry of health officials respective heads	Hand searching

4.	Covid-19	Ministry of health	Ministry of health officials	Hand searching
5.	Diarrheal diseases	Ministry of health	Ministry of health officials	Hand searching
6.	Infectious diseases	Infectious disease society of Kenya	Ministry of health officials<br>Respective head	Hand searching
7.	Cardiovascular diseases	Ministry of health<br>Kenya cardiac association	Ministry of health officials<br>Respective disease head	Hand searching
8.	Cancer	Ministry of health<br>Kenya cancer association<br>National cancer institute of Kenya	Ministry of health officials<br>Respective head	Hand searching

9.	Diabetes mellitus	Ministry of health<br>Kenya diabetes association	Ministry of health officials	Hand searching
9.	Chronic respiratory diseases	Ministry of health	Ministry of health officials	Hand searching
10.	Cirrhosis	Ministry of health	Ministry of health officials	Hand searching
11.	Chronic kidney disease	Ministry of health<br>National kidney foundation<br>Kenya renal association	Ministry of health officials	Hand searching
12.	Nutritional disorders	Ministry of health	Ministry of health officials	Hand searching
13.	Maternal disorders	Ministry of health<br>Kenya obstetrical gynaecological society	Ministry of health officials	Hand searching
14.	Neonatal disorders	Ministry of health<br>Kenya paediatric association	Ministry of health officials	Hand searching

15.	Injuries	Ministry of health	Ministry of health officials	Hand searching
16		Google		Search strategy used-See below

The following search strategy was used to search for CPGs in Google

1.	Clinical practice guidelines
2.	Health guidelines
3.	Clinical guidelines
4.	Guidelines
5.	Clinical protocols
6.	Treatment protocols
7.	Management protocols
8.	1 OR 2 OR 3 AND 26
9.	1 OR 2 OR 3 OR 4 OR 5 OR 6 AND 26
10.	1 OR 2 OR 3 OR 4 OR 5 OR 6 AND 26 AND HIV/AIDS
11.	1 OR 2 OR 3 OR 4 OR 5 OR 6 AND 26 AND Tuberculosis
12.	1 OR 2 OR 3 OR 4 OR 5 OR 6 AND 26 AND Malaria
13.	1 OR 2 OR 3 OR 4 OR 5 OR 6 AND 26 AND Covid-19
14.	1 OR 2 OR 3 OR 4 OR 5 OR 6 AND 26 AND Diarrheal diseases
15.	1 OR 2 OR 3 OR 4 OR 5 OR 6 AND 26 AND Infectious diseases
16.	1 OR 2 OR 3 OR 4 OR 5 OR 6 AND 26 AND Cardiovascular diseases

17.	1 OR 2 OR 3 OR 4 OR 5 OR 6 AND 26 AND Cancer
18.	1 OR 2 OR 3 OR 4 OR 5 OR 6 AND 26 AND Diabetes mellitus
19.	1 OR 2 OR 3 OR 4 OR 5 OR 6 AND 26 AND Chronic respiratory diseases
20.	1 OR 2 OR 3 OR 4 OR 5 OR 6 AND 26 AND Cirrhosis
21.	1 OR 2 OR 3 OR 4 OR 5 OR 6 AND 26 AND Chronic kidney disease
22.	1 OR 2 OR 3 OR 4 OR 5 OR 6 AND 26 AND Nutritional disorders
23.	1 OR 2 OR 3 OR 4 OR 5 OR 6 AND 26 AND Maternal disorders
24.	1 OR 2 OR 3 OR 4 OR 5 OR 6 AND 26 AND Neonatal disorders
25.	1 OR 2 OR 3 OR 4 OR 5 OR 6 AND 26 AND Injuries
26.	Kenya
