# Supplementary material for: Quality assessment of clinical practice guidelines in Kenya using the AGREE II tool: a methodological review
Source: BMJ Open. 2023 Jul 10;13(7):e074510. doi: 10.1136/bmjopen-2023-074510 (PMC10335456; doi:10.1136/bmjopen-2023-074510)
Supplement: Supplementary data [file bmjopen-2023-074510supp003.pdf]

Supplementary file 3: Data extraction form

Items	Description of items	Date of extraction
Title		
Authors		
Year of publication		
Disease domain		
Scope		
