# Supplementary material for: Quality assessment of clinical practice guidelines in Kenya using the AGREE II tool: a methodological review
Source: BMJ Open. 2023 Jul 10;13(7):e074510. doi: 10.1136/bmjopen-2023-074510 (PMC10335456; doi:10.1136/bmjopen-2023-074510)
Supplement: Supplementary data [file bmjopen-2023-074510supp004.pdf]

Supplementary file 4: List of excluded clinical practice guidelines

DISEASE	Name of guideline	AUTHOR	YEAR	SCOPE	Reason for Exclusion
**COVID-19**	COVID-19 guidance on comprehensive HIV service delivery	NASCOP, C&T, MOH	2020	Prevention	Health system CPG
	Interim guidance on provision of services for non communicable diseases (NCDs) during the COVID-19 pandemic	MoH	2020	Management	Interim CPG
	Interim guidance for nutrition and dietetics management of covid-19 for health workers in treatment and isolation centres	MoH	2020	Management	Interim CPG
	Guidelines on continued provision of community health services in the context of corona virus	MoH	2020	Management	health system CPG
	Guidelines on use of antiretroviral drugs for treating and preventing HIV infection in Kenya	MoH & NASCOP	2016	Treatment	More than 5 years
**HIV**	A practical handbook for HIV managers and service providers on differentiated care	NASCOP	2016	Prevention	More than 5 years
	National guidelines for HIV testing and counselling in Kenya	MoH & NASCOP	2008	Diagnosis	More than 5 years
	The Kenya HIV testing services guidelines	MoH & NASCOP	2015	Diagnosis	More than 5 years
	National manual for the management of HIV-related opportunistic infections and conditions	MoH & NASCOP	2008	Integrated	More than 5 years
	National guidelines for PMTCT peer education and psychosocial support in Kenya	MoH	2012	prevention	More than 5 years

	Guidelines for prevention of mother to child transmission (PMTCT) of HIV/AIDS in Kenya	MoH	2012	prevention	More than 5 years
	National guidelines for HIV/STI programming with key populations	MoH	2014	Prevention	More than 5 years
**TB**	Guidelines for implementing TB-HIV collaborative activities in Kenya	MoH	2006	Integrated	More than 5 years
	Guidelines for the management of drug resistant tuberculosis in Kenya	MoH	2010	Management	More than 5 years
	National guidelines for the diagnosis, treatment and prevent of malaria in Kenya	MoH	2010	Integrated	More than 5 years
	National guidelines for the diagnosis, treatment and prevent of malaria in Kenya	MoH	2016	Integrated	More than 5 years
**Malaria**	Guidelines for malaria epidemic preparedness and response in Kenya	MoH	2020	Prevention	Health system CPG
**Cholera**	Policy guidelines for the management of diarrhoea in children below five years in Kenya	MoH	2014	Management	More than 5 years
	Guidelines on cholera control	MoH and WHO	2002	Prevention	More than 5 years
**DM**	National clinical guidelines for management of diabetes mellitus	MoH	2010	Integrated	More than 5 years
	Kenya national diabetes educators manual	MoH	2010	Prevention/management	More than 5 years
**Cancer**	National guidelines for establishment of cancer management centres in Kenya	MoH	2018	Prevention	Health systems
	National cancer specimen handling guidelines		2020	Prevention	not directly on patient care

	National guidelines for cancer management Kenya	MoH	2013	Integrated	More than 5 years
**KIDNEY D**	Guidelines for infection prevention in dialysis units	Kenya renal association		Prevention	More than 5 years
**KIDNEY D**	Policy guidelines on establishment and running of renal dialysis units	MoH	2015	Prevention	More than 5 years
**Maternal**	National guidelines for quality obstetrics and perinatal care	MoH	2016	Integrated	More than 5 years
**neonates/children**	National guidelines on essential newborn care	MoH	2016	Integrated	More than 5 years
	Integrated community case management for sick children under 5 years	MoH	2013	Integrated	More than 5 years
**Nutrition**	National maternal, infant and young child nutrition	MoH	2013	Integrated	More than 5 years
	National guideline for integrated management of acute malnutrition	MoH	2009	Integrated	More than 5 years
	Kenyan national guidelines on nutrition and HIV	MoH	2014	Management	More than 5 years
	Kenya national clinical nutrition and dietetics reference manual	MoH	2010	Integrated	More than 5 years
**injuries**	National guidelines on management of sexual violence in Kenya	MoH	2014	Integrated	More than 5 years
	Occupational safety and health policy guidelines for the health sector in Kenya	MoH	2014	Integrated	More than 5 years

**unclassified**	Clinical guidelines for management and referral of common conditions at level 2 and 3	MoH	2009	Integrated	More than 5 years
	Kenya essential medicines list	MoH	2019	1ntegrated	Health systems CPG

*TB-Tuberculosis

*MoH-Ministry of Health

*NASCOP-National AIDS and STIs Control Program

*HIV-Human Immunodeficiency Virus

*PMTCT- Prevention of Mother To Child Transmission

*WHO-World Health Organization

*STI-Sexually transmitted infection

*NCD-Non-Communicable disease

*COVID-19-Corona virus disease 2019
