# Supplementary material for: Quality assessment of clinical practice guidelines in Kenya using the AGREE II tool: a methodological review
Source: BMJ Open. 2023 Jul 10;13(7):e074510. doi: 10.1136/bmjopen-2023-074510 (PMC10335456; doi:10.1136/bmjopen-2023-074510)
Supplement: Supplementary data [file bmjopen-2023-074510supp005.pdf]

Supplementary file 5: Domain scores

	Domain	Mean percentage	SD	95% CI
**1.**	Scope and purpose	61.75	17.90	[54.19-69.31]
**2.**	Stakeholder involvement	45.25	12.41	[40.01-50.49]
**3.**	Rigour of development	3	5.66	[0.61-5.39]
**4.**	Clarity of presentation	82.96	10.92	[78.35-87.57]
**5.**	Applicability	19.88	15.53	[13.32-26.43]
**5.**	Editorial Independence	6.92	8.17	[3.47-10.37]
