# Supplementary material for: Quality assessment of clinical practice guidelines in Kenya using the AGREE II tool: a methodological review
Source: BMJ Open. 2023 Jul 10;13(7):e074510. doi: 10.1136/bmjopen-2023-074510 (PMC10335456; doi:10.1136/bmjopen-2023-074510)
Supplement: Author's manuscript [file bmjopen-2023-074510.draft_revisions.pdf]

BMJ Open

BMJ Open is committed to open peer review. As part of this commitment we make the peer review history of every article we publish publicly available.

When an article is published we post the peer reviewers' comments and the authors' responses online. We also post the versions of the paper that were used during peer review. These are the versions that the peer review comments apply to.

The versions of the paper that follow are the versions that were submitted during the peer review process. They are not the versions of record or the final published versions. They should not be cited or distributed as the published version of this manuscript.

BMJ Open is an open access journal and the full, final, typeset and author-corrected version of record of the manuscript is available on our site with no access controls, subscription charges or pay-per-view fees (http://bmjopen.bmj.com).

If you have any questions on BMJ Open's open peer review process please email info.bmjopen@bmj.com

BMJ Open

Quality Assessment of Clinical Practice Guidelines in Kenya Using the AGREE II Tool:A Methodological Review

Journal:	*BMJ Open*
Manuscript ID	bmjopen-2023-074510
Article Type:	Original research
Date Submitted by the Author:	08-Apr-2023
Complete List of Authors:	Sagam, Caleb; Kenya Medical Research Institute, Centre for Global Health Research<br>Were, Lisa M.; Kenya Medical Research Institute<br>Otieno, Jenifer A.; Kenya Medical Research Institute<br>Mulaku, Mercy N.; Kenya Medical Research Institute; Stellenbosch University<br>Kariuki , Simon; Kenya Medical Research Institute<br>Ochodo, Eleanor; Kenya Medical Research Institute; Stellenbosch University
Keywords:	Health Equity, Clinical Decision-Making, Health policy < HEALTH SERVICES ADMINISTRATION & MANAGEMENT, Health & safety < HEALTH SERVICES ADMINISTRATION & MANAGEMENT, Protocols & guidelines < HEALTH SERVICES ADMINISTRATION & MANAGEMENT

SCHOLARONE™ Manuscripts

I, the Submitting Author has the right to grant and does grant on behalf of all authors of the Work (as defined in the below author licence), an exclusive licence and/or a non-exclusive licence for contributions from authors who are: i) UK Crown employees; ii) where BMJ has agreed a CC-BY licence shall apply, and/or iii) in accordance with the terms applicable for US Federal Government officers or employees acting as part of their official duties; on a worldwide, perpetual, irrevocable, royalty-free basis to BMJ Publishing Group Ltd ("BMJ") its licensees and where the relevant Journal is co-owned by BMJ to the co-owners of the Journal, to publish the Work in this journal and any other BMJ products and to exploit all rights, as set out in our licence.

The Submitting Author accepts and understands that any supply made under these terms is made by BMJ to the Submitting Author unless you are acting as an employee on behalf of your employer or a postgraduate student of an affiliated institution which is paying any applicable article publishing charge ("APC") for Open Access articles. Where the Submitting Author wishes to make the Work available on an Open Access basis (and intends to pay the relevant APC), the terms of reuse of such Open Access shall be governed by a Creative Commons licence – details of these licences and which Creative Commons licence will apply to this Work are set out in our licence referred to above.

Other than as permitted in any relevant BMJ Author's Self Archiving Policies, I confirm this Work has not been accepted for publication elsewhere, is not being considered for publication elsewhere and does not duplicate material already published. I confirm all authors consent to publication of this Work and authorise the granting of this licence.

For peer review only

Quality Assessment of Clinical Practice Guidelines in Kenya Using the AGREE II Tool: A Methodological Review

*Caleb K. Sagam[1], Lisa M. Were[1], Jenifer A. Otieno[1], Mercy N. Mulaku[1,2,3], Simon Kariuki[1], Eleanor A. Ochodo[1,2]

Author affiliations:

[1]Centre for Global Health Research, Kenya Medical Research Institute, Kisumu, Kenya.

[2]Centre for Evidence-based Health Care, Division of Epidemiology and Biostatistics, Faculty of Medicine and Health Sciences, Stellenbosch University, Cape Town, South Africa.

[3]Department of Pharmacology, Clinical Pharmacy, and Pharmacy Practice, Faculty of Health Sciences, University of Nairobi, Kenya

*Caleb K. Sagam: sagamc7@gmail.com

Lisa M. Were: lisawere1997@gmail.com

Jenifer A. Otieno: jenipherakoth15@gmail.com

Mercy N. Mulaku: mercymulaku@gmail.com

Simon Kariuki: skariuki1578@gmail.com

Eleanor A. Ochodo: eleanor.ochodo@gmail.com

***Corresponding author**: Caleb K. Sagam sagamc7@gmail.com

: P.O. Box 1578-40100, KEMRI-CGHR, Kisumu, Kenya

Word count:3580 (excluding tables, references and cover page)

For peer review only

Abstract

Objective: To assess the quality of available and accessible national Clinical Practice Guidelines (CPGs) in Kenya using the Appraisal of Guidelines for Research and Evaluation II (AGREE II) tool.

Methods: We searched the websites of the Kenyan Ministry of Health, professional associations and contacted experts in relevant organisations. Our scope was guidelines on maternal, neonatal, nutritional disorders, injuries, communicable and non-communicable diseases in Kenya published in the last five years as of 30th June 2022. C.S J.O and L.W independently conducted study selection and data extraction. We conducted a quality assessment using the online English version of AGREE II tool across six domains. Descriptive statistics (scaled domain percentages) were analysed using STATA software version 17 (Stata Corp LLC, College TX. The primary outcome was the methodological quality of the included CPGs assessed by the AGREE II tool score.

Results: We retrieved 95 CPGs and included 24 in the analysis after screening for eligibility. The CPGs scored best in clarity of presentation and least in the rigour of development. In descending order, the appraisal scores (mean and CI) per domain were as follows: Clarity of presentation 82.96% (95% CI 78.35-87.57) with all guidelines scoring above 50%. Scope and purpose 61.75% (95% CI 54.19-69.31) with seven guidelines scoring less than 50%. Stakeholder involvement 45.25% (95% CI 40.01-50.49) with 16 CPGs scoring less than 50%. Applicability domain 19.88% (95% CI 13.32-26.43) with only one CPG scoring above 50%. Editorial independence 6.92% (95% CI 3.47-10.37) with no CPG scoring above 50% and Rigour of development 3% (95% CI 0.61-5.39) with no CPG scoring at least 50%.

Conclusion: Our findings suggest that the quality of CPGs in Kenya is limited mainly by the rigour of development, editorial independence, applicability and stakeholder involvement. Training initiatives on evidence-based methodology among guideline developers are needed to improve the overall quality of CPGs for better patient care.

Keywords: Clinical Practice Guidelines, Quality, AGREE II, Kenya, review.

For peer review only

Strengths and Limitations of this Study

- We used AGREE II instrument, an internationally used and validated tool for the quality of CPGs.
- Three independent reviewers rigorously appraised the CPGs and the final score was reached through a consensus
- Poor reporting in identified CPGs limited our assessment with the AGREE II tool.

Protocol Preprint DOI: https://doi.org/10.31219/osf.io/3nu2q

Funding sources

This study is funded under the UK MRC African Research Leaders award (MR/T008768/1). This award is jointly funded by the UK Medical Research Council (MRC) and the UK Foreign, Commonwealth & Development Office (FCDO) under the MRC/FCDO Concordat agreement. It is also part of the EDCTP2 programme supported by the European Union."

Conflict of interest

None declared.

For peer review only

Introduction

Clinical Practice Guidelines (CPGs) aid healthcare professionals in decision-making and provide a tool to implement recommended health policies at the point of care (1). According to the United States of America Institute of Medicine, CPGs are systematically developed recommendations that help healthcare practitioners make appropriate healthcare decisions to optimise patient care, informed by a systematic review of the evidence, assessment of benefits, harms and alternative care options (2). Good quality evidence-based guidelines developed or adapted robustly and transparently are essential in ensuring good quality healthcare (3).

Kenya, a low-middle income country with a life expectancy of about 66.7 years, is faced with health challenges, including a high burden of communicable diseases, an increasing burden of non-communicable diseases (NCDs), a high maternal & child mortality and injuries (4). Communicable diseases have remained the leading cause of morbidity and mortality (5). In 2020, mortality due to communicable diseases in the general population was 50.1%, a decline compared to 58.6% in 2016 (6). Mortality due to NCDs is on the rise, with 42.8% of deaths being attributed to NCDs in 2020, compared to 37% in 2016, while mortality attributed to injuries was 7.1% in 2020 (4). Four major NCDs, including cancer, diabetes, chronic respiratory and cardiovascular diseases, account for 33% of all the NCD deaths in Kenya, with other diseases such as mental health conditions on the increase (7). Mortality is mainly high among infants and young children and those above the age of 65 years, a trend similar in both males and females (6). According to High-Quality Technical Assistance for Results (HEART) organisation 2018 annual report, maternal and neonatal mortalities in Kenya account for 360/100,000 and 22/1000 live births, respectively (8).

A skilled and well-guided health workforce is needed to tackle the outlined disease burden in Kenya. The World Health Organization (WHO) defines core healthcare workers as medical doctors, nurses, and clinical officers/doctors assistants and recommends a minimum core health worker density threshold of 23 health workers per 10,000 population for effective service delivery (9, 10). As of 2020, Kenya's core health worker density was 16.6 health workers per 10,000, with 1.7 doctors per 10,000 population and 12.1 nurses per 10,000 population (6). With a shortage of health workers amidst a high disease burden,

For peer review only

well-developed, implemented, and accessible CPGs are essential in guiding quality healthcare practices and improving the quality of life.

Previous studies have shown that CPGs improve health outcomes (11). In a study by Flarity *et al*, CPGs were found to reduce hospital length of stay (12). According to Ruseckaite *et al,* the use of CPGs in Australia led to an improvement in the nutritional status of children with cystic fibrosis (13). Implementation strategies of the CPGs have also been shown to improve health outcomes (3). An assessment of an international cohort of CPGs for glycaemic control in patients with type 2 diabetes mellitus highlighted the variation in the quality of CPGs and their effect on the quality of care (14). To our knowledge, this is the first evaluation of the methodological quality of CPGs in Kenya. We aimed to assess the methodological quality of available and accessible national CPGs in Kenya using the Appraisal of Guidelines for Research & Evaluation (AGREE II) tool (15); (Supplementary file 1). This review will inform the future development of high-quality evidence-based guidelines and recommend areas of strengthening the current CPGs in Kenya and beyond.

Methods

Eligibility criteria

Data on mortality and morbidity in Kenya are sparse and the available data are often not linked together to produce a comprehensive ranking of the disease burden. We, therefore, included available and accessible Kenyan CPGs on diseases or conditions under the broad health categories identified by the global burden of diseases, injuries, and risk factors study 2019 (16). The broad health categories included communicable diseases, non-communicable diseases, maternal disorders, neonatal disorders, nutritional disorders, and injuries. We included the latest CPGs versions for screening, diagnosis, and treatment/management used in direct patient care for adults and children. We included CPGs published in English in the last five years as of 30th June 2022. It is estimated that CPGs get outdated five years after their development (1).

We excluded guideline reports (manuscripts), summaries, press statements, or interim guidance documents because they could have incomplete evidence or guidance. We also excluded health system guidelines because they are not used directly in patient care.

For peer review only

Identification of clinical guidelines

We searched the websites of the Kenyan Ministry of Health, Health Associations and contacted relevant organisations' personnel for additional guidelines. We also searched the Kenya e-repository (for Kenyan government documents). Using Google, we searched, the following search terms "clinical practice guidelines," "health guidelines," and "Kenya". A detailed list of websites and databases can be found in supplementary file 2.

Screening and selection

We conducted CPG's screening and selection using an Excel spreadsheet, where we arranged all retrieved guidelines according to diseases and the latest guideline for each disease was picked for appraisal. C.S, J.O and L.W screened the title and publication year of CPG documents for eligibility. We retrieved CPGs that meet the eligibility criteria for quality assessment. We resolved discrepancies on inclusion through consensus or discussion with E.O. Selection process details are provided in the Preferred Reporting Items for Systematic Reviews and Meta-Analysis 2020 (PRISMA) flow chart below (17). The protocol for this review was registered in Open Science Framework (OSF) (18).

Data extraction

C.S, J.O and L.W independently extracted data using a Google form into an Excel worksheet (supplementary file 3). We extracted the general information of the included guidelines, including title, year of publication, author, disease, and scope of the guideline. We resolved discrepancies through consensus or discussion with E.O.

Quality assessment

C.S, J.O and L.W independently assessed the quality of CPGs using the AGREE II tool, a validated and widely used tool, recommended by WHO for assessing the quality, transparency and rigour of CPGs (15). An international group of guideline developers and researchers first developed AGREE II in 2003. It was published in 2009, revised in 2017, and is currently used globally in appraising CPGs (19). It contains six domains and 23 items (15). The six domains evaluated include scope and purpose, stakeholder involvement, the rigour of development, (evidence-based methodology), clarity of presentation, applicability and editorial independence (conflict of interest).

For peer review only

The reviewers conducted the appraisal and scored each item against a seven-point Likert scale on the online version of AGREE II. We scored one if none of the criteria for an item was met or the item was reported very poorly. We scored seven when the item met all the criteria and was well-reported.

The primary outcome of this study was the methodological quality of the CPGs assessed by AGREE II score. The quality scores were determined by calculating the scaled domain percentages for each CPG as outlined in the AGREE II tool (15). We have reported each item's average appraiser score and overall assessment scores. We also calculated the average scores for the 23 items and the overall assessment score for each CPG using STATA software version 17 (Stata Corp LLC, College TX). We present our results in tables as means, confidence intervals, standard deviations and graphs. The overall assessment score is not an average of individual domain scores but an independent domain.

Results

Search results

We retrieved 95 CPGs from the Ministry of Health Kenya, different divisions and health associations' websites. After the removal of duplicates, we identified 61 guidelines. During full-text screening, we excluded 37 guidelines that did not meet the inclusion criteria. We finally included 24 CPGs (figure 1). A list of excluded studies can be found in supplementary file 4.

General characteristics of included guidelines

The description of the general characteristics of the assessed guidelines is illustrated in (Table 1). Most of the guidelines were integrated (these are guidelines containing more than one purpose i.e., Diagnosis and screening and treatment or management) (n=19 CPGs). Six CPGs were published in 2017, six in 2018, two in 2019, five in 2020, four in 2021, and one in 2022. The Ministry of Health Kenya authored majority of the guidelines.

For peer review only

Table 1: Characteristics of included clinical practice guidelines

Disease domain	Title of the guideline	Author	Year of publication	Scope
COVID-19	Guidelines on case management of COVID-19 in Kenya (20)	MoH	2021	Integrated
	Guidelines to be used by occupational therapists in rehabilitation of patients during COVID-19 pandemic (18)	MoH	2020	Management
HIV	Guidelines on use of antiretroviral drugs for treating and preventing HIV infection in Kenya (21)	MoH & NASCOP	2018	Integrated
	National guidance on tetanus prevention in voluntary medical male circumcision settings in Kenya (22)	MoH	2017	Prevention
TB	National guidelines on management of tuberculosis in children (23)	MoH	2017	Integrated
	Integrated guideline for tuberculosis, leprosy and lung disease (24)	MoH	2021	Integrated
	Field guide on systematic screening of active TB in Kenya (25)	MoH	2017	Screening
Malaria	Guidelines for the diagnosis, treatment & prevention of malaria in Kenya (26)	MoH	2020	Integrated
CVD	Kenya national guidelines for cardiovascular diseases management (27)	MoH	2018	Integrated
Eye	National guidelines for the management of glaucoma (28)	MoH	2020	Integrated
	Guidelines for the screening and management of retinopathy of prematurity in Kenya (29)	MoH	2018	Integrated
Diabetes	National clinical guidelines for management of diabetes mellitus (30)	MoH	2018	Integrated
	Guidelines for screening and management of diabetic retinopathy (31)	MoH	2017	Integrated

For peer review only

Cancer	Kenya national cancer treatment protocols (32)	MoH	2019	Integrated
	Retinoblastoma best practice guidelines (33)	MoH	2019	Integrated
	National cancer screening guidelines (34)	MoH	2018	Screening
Renal disease	Guidelines for the management of emergencies in dialysis (35)	Kenya renal association	2017	Management
Mental health	National tele-mental health guidelines (36)	MoH	2021	Integrated
Respiratory diseases	Kenya asthma management guideline (37)	MoH	2021	Integrated
Maternal health	Guidelines for postnatal care (38)	MoH	2017	Integrated
Paediatrics	Guidelines on the management of paediatric patients during COVID-19 pandemic (39)	MoH	2020	Integrated
	Basic paediatric protocols(40)	MoH	2022	Integrated
Sickle Cell	National guidelines for control and management of sickle cell disease in Kenya (41)	MoH	2020	Integrated
Nutrition	National guidelines for healthy diets and physical activity (42)	MoH	2017	Prevention

*MoH-Ministry of Health

*NASCOP-National AIDS and STIs Control Program

*HIV-Human Immunodeficiency Virus

*TB-Tuberculosis

*CVD-Cardiovascular diseases

For peer review only

Findings on AGREE II guideline quality score per domain

In reference to Table 2, Figure 2 and Supplementary file 5, we present the findings from the highest-scoring domain to the least-scoring domain:

Clarity of presentation

The mean score was 82.96% (95% CI 78.35-87.57) with all guidelines scoring above 50%. The overall mean of this domain was the highest among all domains. Recommendations were specific and unambiguous. Key recommendations were easily identifiable and different options for managing the health condition were stated in varying ways. Items 15 and 16 scored above six and 17 scored above five (Table 3).

Scope and purpose

The mean score in this domain was 61.75% (95% CI 54.19-69.31). Most guidelines scored well in this domain with only seven scoring less than 50%. Under domain one items one, two and three scored above four out of the possible seven. This is because the health questions, the guideline objective and the population to whom the guideline was applied were described in most guidelines. However, adequate information was not reported on the expected benefit or outcome and health care setting. The description of health intents was not well written and clear in some guidelines.

Stakeholder involvement

The mean score in this domain was 45.25% (95% CI 40.01-50.49). This domain score was lower than domain one because most guidelines did not involve evidence-based experts and patients in the development process. Sixteen CPGs scored less than 50%. In domain two, items four and six scored above four out of possible seven (Table 3). Item five had a score of one, this is because the views and preferences of the target population were only sought in 2 out of the 24 CPGs. Disciplines and a description of the member's role in the guideline development group were missing in most guidelines.

Applicability

The applicability domain mean score was 19.88% (95% CI 13.32-26.43) with only one CPG scoring above 50%. Most of the CPGs did not describe the facilitators and barriers of its application or provide advice on how tools can be put into practice. They also did not

For peer review only

present monitoring and evaluation criteria and potential resource implications of the recommendations. Under domain five, all the items scored below three out of the possible seven, especially in items 18 and 20 (Table 3).

Editorial independence

The mean score was 6.92% (95% CI 3.47-10.37) with no CPG scoring above 50%. The scores in this domain were the second lowest after the rigour of development. Items in this domain scored less than two, this is because information on funding was either not stated or not clear. Conflict of interest was poorly reported, this contributed to the low score in domain six because information on the declaration of competing interest by the guideline development group was not provided. Though a third of the guidelines mentioned the funding bodies, the role of the funding bodies in the guideline development process was not provided.

Rigour of development

This domain has the highest number of items and plays a key role in developing CPGs. The mean was 3% (95% CI 0.61-5.39) with no CPG scoring at least 50%. The overall domain score was poor with the highest score of 26%. A total of eight CPGs scored zero percent in this domain which points out serious shortcomings.

Under domain three, all the items from items seven to 14 scored less than two out of the possible seven (Table 3). Systematic methods were not used to search for evidence, the criteria for selecting the evidence were not described and the strengths and limitations of the evidence were not described. In addition, methods for formulating recommendations were not clear, there was no information on whether health benefits, side effects, harms and risks were considered in formulating recommendations. An explicit link between recommendation and supporting evidence was also not presented and there was no information to show that the guideline was externally reviewed before its publication. All these factors led to a score of less than two for each item.

In this study, the scores of the 24 CPGs on the six domains, the only two domains scored above 50% is scope and purpose and clarity of presentation. These guidelines scored low in editorial independence, applicability and rigour of development.

For peer review only

Table 2: Domain scores for each CPG

CPG Name	Domain percentages scores						Average
	1	2	3	4	5	6	(%)
Guidelines for screening and management of diabetic retinopathy (31)	96	72	26	81	42	28	67
National guidelines for the management of glaucoma (28)	98	63	11	96	29	0	61
Integrated guideline for tuberculosis, leprosy and lung disease (24)	67	56	1	93	42	8	56
Guidelines for the screening and management of retinopathy of prematurity in Kenya (29)	81	63	5	91	31	8	56
National guidelines for healthy diets and physical activity (42)	81	44	0	94	53	3	56
Field guide on systematic screening of active TB in Kenya (25)	56	30	1	89	49	0	50
National cancer screening guidelines (34)	70	54	2	94	7	11	50
Kenya asthma management guideline (37)	78	52	1	87	38	3	50
National guidelines for control and management of sickle cell disease in Kenya (41)	70	54	1	85	13	0	50
National guidance on tetanus prevention in voluntary medical male circumcision settings in Kenya (22)	44	30	8	72	29	17	44
National guidelines on management of tuberculosis in children (23)	69	52	0	76	19	0	44
Guidelines for the diagnosis, treatment & prevention of malaria in Kenya (26)	70	44	1	91	14	14	44
Kenya national cancer treatment protocols (32)	44	50	0	94	11	6	44
Retinoblastoma best practice guidelines (33)	54	41	4	85	8	3	44
Guidelines on the management of pediatric patients during COVID-19* pandemic (39)	57	28	6	80	0	0	44
National clinical guidelines for management of diabetes mellitus (30)	63	46	0	83	11	25	44
Basic pediatric protocols (40)	50	41	1	78	18	6	44

For peer review only

Guidelines to be used by occupational therapists in rehabilitation of patients during covid-19* pandemic (18)	65	52	2	80	7	0	39
Kenya national guidelines for cardiovascular diseases management (27)	37	37	0	93	14	6	39
Guidelines for postnatal care (38)	65	48	0	78	17	3	39
Guidelines on case management of COVID-19* in Kenya (20)	56	30	1	81	4	0	33
Guidelines on the use of antiretroviral drugs for treating and preventing HIV infection in Kenya (21)	48	44	0	80	7	19	33
National tele-mental health guidelines (36)	35	31	0	54	14	6	33
Guidelines for management of emergencies in dialysis (35)	28	24	1	56	0	0	28

*TB-Tuberculosis

*HIV-Human Immunodeficiency Virus

*COVID-19-Corona virus disease 2019

For peer review only

Table 3: Quality of CPGs per item of the AGREE II tool

Variable	Mean	Std. dev.	[95% conf. interval]
Item 1	5.03	1.43	[4.42 5.63]
Item 2	4.06	1.57	[3.39 4.72]
Item 3	4.54	1.25	[4.01 5.07]
Item 4	4.26	1.35	[3.69 4.84]
Item 5	1.32	0.68	[1.03 1.61]
Item 6	5.66	1.49	[4.93 6.18]
Item 7	1.13	0.35	[0.98 1.27]
Item 8	1.07	0.22	[0.98 1.16]
Item 9	1.01	0.07	[0.99 1.04]
Item 10	1.11	0.29	[0.99 1.23]
Item 11	1.08	0.28	[0.96 1.20]
Item 12	1.24	0.71	[0.94 1.54]
Item 13	1.14	0.37	[0.98 1.29]
Item 14	1.68	1.35	[1.11 2.25]
Item 15	6.10	0.79	[5.76 6.43]
Item 16	6.32	0.75	[6.00 6.63]
Item 17	5.61	0.95	[5.21 6.01]
Item 18	1.61	0.93	[1.22 2.00]
Item 19	2.94	0.98	[2.53 3.36]
Item 20	1.49	0.85	[1.13 1.85]
Item 21	2.71	1.95	[1.89 3.53]
Item 22	1.82	0.98	[1.40 2.23]
Item 23	1.00	0.00	[1.00 1.00]
Overall assessment	3.74	0.56	[3.50 3.97]

For peer review only

DISCUSSION

We assessed the quality of 24 CPGs in Kenya published in the last five years as of 30th June 2022 using the AGREE II tool across six domains: clarity of presentation, scope and purpose, stakeholder involvement, applicability, editorial independence and rigour of development. The CPGs scored best in clarity of presentation (82.96% (95% CI 78.35-87.57)) and scored least in the rigour of development (3% (95% CI 0.61-5.39)).

Similar to our results, a study done in China reported challenges in the rigour of development (43). The quality of CPGs is heavily affected by the use of evidence-based methods (44). In a systematic review of the effect of evidence-based CPGs on the quality of care, evidence-based clinical guidelines improved the quality of care (3). It is recommended that guideline recommendations be made based on the best available evidence using an evidence-to-decision framework (EtD) (45). EtD frameworks (such as the Grading of Recommendations, Assessments and Evaluations (GRADE EtD) and WHO INTEGRATE (EtD) provide a systematic and explicit process linking the best available evidence to formulating the recommendations (46-48).

According to a study on publishing CPGs, external review plays a critical role in enhancing scientific validity and clarity but also in enhancing scientific validity, clarity and feasibility of the guidelines before formal publication (49). External review is a critical step in enhancing accountability during guideline development. It provides an opportunity for additional inputs and a critical review of the guideline by a specialist before its publication. Our findings show that over two-thirds of the guidelines did not report on the procedure and frequency of updating the guideline. The body of knowledge is constantly changing, therefore there is a need to review CPGs. Updating CPGs is very important as it enhances the validity of the recommendations (50). According to a systematic review of guidance for updating CPGs two to three years is the recommended time frame between publishing a guideline and commencing the updating process (51).

Similar to our results, a study done in China found shortcomings in editorial independence (43). This can be attributed to conflict of interest disclosure policies and whether they have been developed or challenges that come with implementation. Transparency is a key aspect in the development of CPGs (49). According to a study on reporting financial

For peer review only

conflicts of interest in CPGs, conflict of interest is a threat because adequate literature is documented on the influence of uncontrolled conflict of interest on recommendations (52). The influence of external activities such as grant funding and ownership of commercial entities have created a bias and affected the process of making recommendations (53). Efforts to balance guideline development groups (GDGs) have improved however included experts in the industry involved in commercial activities which makes it difficult to rule out a conflict of interest (49). Reporting is also an integral part of ensuring transparency, because when a conflict of interest was done but not reported it brings confusion and it will always be assumed that it was not done which may not be the reality (54).

Poor applicability threatens the usability of the guidelines at the facility level. The applicability domain goes beyond the methodological quality and covers resource implication, which is key to successfully implementing each guideline (55). Similar to our study on appraisal of clinical guidelines for recurrent urinary tract infections also showed low scores in this domain (51). Monitoring and evaluation plan ensures the guidelines serve their intended purpose. In many cases the guideline developers may not be responsible for the direct implementation of the guideline therefore it is important to stipulate how the monitoring and auditing will be done to ensure adequate implementation of the guideline.

GDG composition is one of the most important aspects of guideline development (48). A GDG necessitates the expertise of healthcare professionals, patient input on their needs and preferences and methodologists and librarians skilled in gathering, summarising, and interpreting evidence (56). Structured education in this methodological area will be required, given the increasing demands and expertise required to manage complex guideline development projects (57). The focus of the guidelines should influence the number of group members and the balance of disciplines. When deciding on the group's composition, all potential stakeholders should be identified, including healthcare professionals who are directly involved in the clinical management of patients in various healthcare settings (for example, primary and secondary care), policymakers who may need to make resource utilisation decisions and patients (57). The decision must then be made about which categories of participants to include in the guideline group. Guidelines

For peer review only

developers must frequently balance the desire for broad representation with the need for a cohesive working group. Small groups may lack experience among their members, while larger groups may lack cohesiveness and be difficult to lead (58).

Implication for practice

Given our findings, we recommend that awareness of the Reporting Items for Practice Guidelines in HealThcare (RIGHT) statement among guideline developers be improved (59). This could be done through conferences, webinars and training workshops by increasingly visible guideline or evidence-based groups in Africa (such as Guidelines International Network(60, 61), Cochrane Africa (62, 63), Africa Evidence Network (64), Joanna Briggs Institute (65, 66) and GRADE networks in Africa (67)). Increased awareness will enable better and more transparent reporting of CPGs. More training initiatives among GDG members on aspects of AGREE II and RIGHT guidelines and the involvement of a guideline methodologist will improve the rigour of development, reporting and overall methodological quality of CPGs in Africa.

Implications for research

A qualitative study to explore guideline developers' knowledge and perspectives on guideline development methods would be useful in knowing the barriers and enablers to guideline development in low and middle-income countries (LMIC). Qualitative research among the users of the guidelines (healthcare workers) would also shed light on barriers to the impact of the guidelines on health practices and outcomes. Impact evaluations of the effect of low- or high-quality CPGs on healthcare in LMIC settings are also needed.

Conclusion

The quality of CPGs in Kenya is limited mainly by the rigour of development, editorial independence, applicability and stakeholder involvement. Training initiatives on evidence-based methodology among guideline developers are needed to improve the overall quality of CPGs for better patient care.

Ethics approval

For peer review only

We did not seek formal ethical approval since this is a methodological review based on publicly available CPGs.

Author Contributions

Conceptualisation of the study: E.O.

Writing of the first draft: C.S., J.O., and L.W.

Critical review of the manuscript J.O., L.W., M.M., S.K, and E.O.

Writing the final draft of the manuscript CS.

Approval of the final draft of the manuscript: C.S., L.W., J.O., M.M., S.K., E.O.

References

1. Gary S. Gronseth, David Gloss, Julie Cox, et al. American Academy of Neurology Clinical Practice Guideline Process Manual.: American Academy of Neurology; 2017.
2. Graham R MM, Miller Wolman D, Greenfield S, et al. Clinical Practice Guidelines We Can Trust. Institute of Medicine (US) Committee on Standards for Developing Trustworthy Clinical Practice Guidelines, Washington (DC): National Academies Press (US); 2011.
3. Lugtenberg M, Burgers JS, Westert GP. Effects of evidence-based clinical practice guidelines on quality of care: a systematic review. Qual Saf Health Care. 2009;18(5):385-392. doi:10.1136/qshc.2008.028043
4. MoH. Kenya Health Sector Strategic Plan, June 2018-2023 Transforming Health Systems: Achieving Universal Health Coverage by 2022. Nairobi: Ministry of Health, Kenya,2018. Available from: https://www.health.go.ke/wp-content/uploads/2020/11/Kenya-Health-Sector-Strategic-Plan-2018-231.pdf, accessed 20th April 2022.
5. Bigna JJ, Noubiap JJ. The rising burden of non-communicable diseases in sub-Saharan Africa. Lancet Glob Health. 2019;7(10): e1295-e1296. doi:10.1016/S2214-109X (19)30370-5
6. MoH. Kenya Health Sector strategic plan 2018-2023, mid-term review synthesis report. Monitoring Progress Towards Attainment of Universal Health Coverage. Nairobi: Ministry of Health, Kenya, 2021. Available from: https://www.health.go.ke/wp-content/uploads/2022/01/KHSSP-MTR-Syntheis-Report2021.pdf, accessed 20th April 2022.

For peer review only

7. MoH. Kenya National Strategy for the Prevention and Control of Non-Communicable Diseases 2015 - 2020. Nairobi: Ministry of Health, Kenya, 2015. Available from: https://www.worlddiabetesfoundation.org/sites/default/files/Kenya%20national%20strategy%20for%20NCDs%202015-2020%20%281%29.pdf, accessed 20th April 2022.
8. Results H-QTAf. Reducing Maternal and Neonatal Deaths in Kenya. Annual Review – post-April 2018.; 2018. Available from: https://www.heart-resources.org/wp-content/uploads/2018/12/MNH_Programme_2018_Annual_Review.pdf, accessed 30th April 2022.
9. WHO. Monitoring the building blocks of health systems. A Handbook of Indicators and their measurement strategies 2010. Geneva: World Health Organization. Available from:https://apps.who.int/iris/bitstream/handle/10665/258734/9789241564052-eng.pdf, accessed 20th April 2022.
10. WHO. Global strategy on human resources for health workforce 2030: Geneva: World Health Organization 2016. Available from: https://apps.who.int/iris/bitstream/handle/10665/250368/9789241511131-eng.pdf. accessed 20th April 2022.
11. Grimshaw JM, Russell IT. Effect of clinical guidelines on medical practice: a systematic review of rigorous evaluations. Lancet. 1993;342(8883):1317-1322. doi:10.1016/0140-6736(93)92244-n
12. Flarity K, Rhodes WC, Berson AJ, et al. Guideline-Driven Care Improves Outcomes in Patients with Traumatic Rib Fractures. Am Surg. 2017;83(9):1012-1017. PMID: 28958283
13. Ruseckaite R, Pekin N, King S, et al. Evaluating the impact of 2006 Australasian Clinical Practice Guidelines for nutrition in children with cystic fibrosis in Australia. Respir Med. 2018;142:7-14. doi:10.1016/j.rmed.2018.07.007
14. Holmer HK, Ogden LA, Burda BU, et al. Quality of clinical practice guidelines for glycemic control in type 2 diabetes mellitus. PLoS One. 2013;8(4):e58625. doi:10.1371/journal.pone.0058625
15. Brouwers MC, Kho ME, Browman GP, et al. AGREE II: advancing guideline development, reporting and evaluation in health care. CMAJ. 2010;182(18):E839-E842. doi:10.1503/cmaj.090449
16. GBD 2019 Diseases and Injuries Collaborators. Global burden of 369 diseases and injuries in 204 countries and territories, 1990-2019: a systematic analysis for the

For peer review only

Global Burden of Disease Study 2019. Lancet. 2020;396(10258):1204-22. PMID: 33069326 PMCID: PMC7567026 DOI: 10.1016/S0140-6736(20)30925-9

17. Page MJ, McKenzie JE, Bossuyt PM, et al. The PRISMA 2020 statement: an updated guideline for reporting systematic reviews. BMJ. 2021;372: n71. Published 2021 Mar 29. PMID: 33782057 PMCID: PMC8005924 DOI: 10.1136/bmj. n71
18. Sagam CK, Were L, Otieno JA, et al. Quality Assessment of Clinical Practice Guidelines in Kenya Using the AGREE II Tool: Methodological Review Protocol https://osf.io/preprints/3nu2q/.DOI: 10.31219/osf.io/3nu2q
19. Brouwers MC, Kho ME, Browman GP, et al. AGREE-II-Users-Manual-and-23-item-Instrument-2009-Update. 2017. Available at: https://www.agreetrust.org/wp-content/uploads/2017/12/AGREE-II-Users-Manual-and-23-item-Instrument-2009-Update-2017.pdf
20. MoH. Guidelines on Case Management of COVID-19 in Kenya. Nairobi: Ministry of Health, Kenya, 2021. Available at: https://www.health.go.ke/wp-content/uploads/2021/10/Final-guidelines-on-the-Management-of-Covid-19-in-Kenya-2021-Edition.pdf, accessed 20th April 2022.
21. MoH. Guidelines on Use of Antiretroviral Drugs for Treating and Preventing HIV Infection in Kenya. Nairobi: Ministry of Health, Kenya, 2018. Available from: https://www.nascop.or.ke/new-guidelines/, accessed 20th April 2022.
22. MoH. National guidance on tetanus prevention in voluntary medical male circumcision settings in Kenya. Nairobi: Ministry of Health, Kenya, 2017. Available from: https://www.nascop.or.ke/new-guidelines/, accessed 20th April 2022.
23. MoH. National Guidelines on Management of Tuberculosis in Children. Nairobi: Ministry of Health, Kenya, 2017. Available from: https://www.nltp.co.ke/guideline/, accessed 20th April 2022.
24. MoH. Integrated Guideline for Tuberculosis, Leprosy and Lung Disease. Nairobi: Ministry of Health, Kenya, 2021. Available from: https://www.nltp.co.ke/guideline/, accessed 20th April 2022.
25. MoH. Field guide on systematic screening of active TB in Kenya. Nairobi: Ministry of Health, Kenya, 2017. Available from: https://chskenya.org/wp-content/uploads/2017/09/Active-Case-Finding-Booklet-1.pdf, accessed 20th April 2022.
26. MoH. Guidelines for the diagnosis, treatment and prevention of malaria in Kenya. Nairobi: Ministry of Health, Kenya, 2020.

For peer review only

27. MoH. Kenya National Guidelines for Cardiovascular Diseases Management. Nairobi: Ministry of Health, Kenya 2018. Available from: http://guidelines.health.go.ke:8000/media/Kenya_National_Guidelines_for_Cardiovascular_Diseases_Management.pdf, accessed 20th April 2022.
28. MoH. National guidelines for the management of Glaucoma. Nairobi: Ministry of Health, Kenya, 2020. Available from: https://www.health.go.ke/wp-content/uploads/2020/12/National-Guidelines-for-Management-of-Glaucoma-Nov.2020.pdf, accessed 20th April 2022.
29. MoH. National guidelines for screening and management of retinopathy of prematurity. Nairobi: Ministry of Health, Kenya, 2018. Available from: https://www.health.go.ke/wp-content/uploads/2021/02/National-Guidelines-for-Screening-and-Management-of-Retinopathy-of-Prematurity.2018.pdf, accessed 20th April 2022.
30. MoH. Kenya National Clinical Guidelines for Management of Diabetes Mellitus. Nairobi: Ministry of Health, Kenya, 2018.
31. MoH. Guidelines for Screening and Management of Diabetic Retinopathy. Nairobi: Ministry of Health, Kenya, 2017. Available from: https://www.health.go.ke/wp-content/uploads/2017/11/Guidelines-for-Screening-and-Management-of-Diabetic-Retinopathy-in-Kenya.pdf, accessed 20th April 2022.
32. MoH. Kenya National Cancer Treatment Protocols. Nairobi: Ministry of Health, Kenya, 2019. Available from: https://www.health.go.ke/wp-content/uploads/2019/09/National-treatment-Protocols-2019.pdf, accessed 20th April 2022
33. MoH. Retinoblastoma-Best-Practice-Guidelines. Nairobi: Ministry of Health, Kenya, 2019. Available from: https://www.health.go.ke/wp-content/uploads/2021/02/RETINOBLASTOMA-Best-Practice-Guidelines-2019.pdf, accessed 20th April 2022.
34. MoH. Kenya National Cancer Screening Guidelines. Nairobi: Ministry of Health, Kenya, 2018. Available from: https://www.health.go.ke/wp-content/uploads/2019/02/National-Cancer-Screening-Guidelines-2018.pdf, accessed 20th April 2022.
35. MoH. Guidelines for the management of emergencies in dialysis. Nairobi: Ministry of Health, Kenya, 2017. Available from: accessed 20th April 2022.

For peer review only

36. MoH. National Tele Mental-Health Guidelines. Nairobi: Ministry of Health, Kenya, 2021. Available from: https://www.health.go.ke/wp-content/uploads/2023/02/MOH-TELE-MENTAL-HEALTH-GUIDELINESUpdated-1.pdf, accessed 20th April 2022.
37. MoH. Kenya Asthma Management Guideline. Nairobi: Ministry of Health, Kenya, 2021. Available from: https://www.nltp.co.ke/guideline/, accessed 20th April 2022.
38. MoH. Guidelines for Postnatal Care. Nairobi: Ministry of Health, Kenya, 2017. Available from: https://familyhealth.go.ke/wp-content/uploads/2018/02/Guidelines-for-postnatal-care-to-mothers-and-newborns-2016-18-12-2016B.pdf,accessed 20th April 2022.
39. MoH. Guidelines for Management of Pediatric Patients During COVID-19 Pandemic. Nairobi: Ministry of Health, Kenya, 2020. Available from: https://www.health.go.ke/wp-content/uploads/2020/06/PAEDIATRIC-Covid-Guidelines-Final.pdf, accessed 20th April 2022.
40. MoH. Basic pediatric protocol 5th edition. Nairobi: Ministry of Health, Kenya, 2022. Available from: https://www.coursehero.com/file/134237800/Basic-Paediatric-Protocols-JAN-27-2022-DRAFT-SWpdf/ accessed 20th April 2022.
41. MoH. National Guidelines for the Management of Sickle Cell Disease in Kenya. Nairobi: Ministry of Health, Kenya, 2020. Available from: https://ncdak.org/wp-content/uploads/2021/07/NATIONAL-GUIDELINES-FOR-CONTROL-AND-MANAGEMENT-OF-SICKLE-CELL-DISEASE-IN-KENYA-.pdf, accessed 20th April 2022.
42. MoH. National Guidelines for Healthy Diets and Physical Activity. Nairobi: Ministry of Health, Kenya, 2017. Available from: http://www.nutritionhealth.or.ke/wp-content/uploads/Downloads/National%20Guidelines%20for%20Healthy%20Diets%20and%20Physical%20Activity%202017.pdf, accessed 20th April 2022.
43. Gao Y, Wang J, Luo X, et al. Quality appraisal of clinical practice guidelines for diabetes mellitus published in China between 2007 and 2017 using the AGREE II instrument. BMJ Open. 2019;9(9):e022392. Published 2019 Sep 4. doi:10.1136/bmjopen-2018-022392
44. Graham ID, Harrison MB. Evaluation and adaptation of clinical practice guidelines. Evid Based Nurs. 2005;8(3):68-72. doi:10.1136/ebn.8.3.68
45. Li SA, Yousefi-Nooraie R, Guyatt G, et al. A few panel members dominated guideline development meeting discussions: Social network analysis. *J Clin Epidemiol*. 2022;141:1-10. doi:10.1016/j.jclinepi.2021.09.023

For peer review only

46. Alonso-Coello P, Schünemann HJ, Moberg J, et al. GRADE Evidence to Decision (EtD) frameworks: a systematic and transparent approach to making well informed healthcare choices. 1: Introduction. BMJ. 2016;353:i2016. Published 2016 Jun 28. doi:10.1136/bmj.i2016
47. Rehfuess EA, Stratil JM, Scheel IB, et al. The WHO-INTEGRATE evidence to decision framework version 1.0: integrating WHO norms and values and a complexity perspective. *BMJ Glob Health*. 2019;4(Suppl 1):e000844. Published 2019 Jan 25. doi:10.1136/bmjgh-2018-000844
48. Atkins D, Best D, Briss PA, et al. Grading quality of evidence and strength of recommendations. BMJ. 2004;328(7454):1490. doi:10.1136/bmj.328.7454.1490
49. Yang N, Zhao W, Qi W, et al. Publishing clinical prActice GuidelinEs (PAGE): Recommendations from editors and reviewers. J Evid Based Med. 2022;15(2):136-141. doi:10.1111/jebm.12479
50. Smith CA, Toupin-April K, Jutai JW, et al. A Systematic Critical Appraisal of Clinical Practice Guidelines in Juvenile Idiopathic Arthritis Using the Appraisal of Guidelines for Research and Evaluation II (AGREE II) Instrument. PLoS One. 2015;10(9):e0137180. Published 2015 Sep 10. doi:10.1371/journal.pone.0137180
51. Pat JJ, Witte LPW, Steffens MG, et al. Quality appraisal of clinical guidelines for recurrent urinary tract infections using AGREE II: a systematic review. Int Urogynecol J. 2022;33(5):1059-1070. doi:10.1007/s00192-022-05089-6
52. Shnier A, Lexchin J, Romero M, et al. Reporting of financial conflicts of interest in clinical practice guidelines: a case study analysis of guidelines from the Canadian Medical Association Infobase. BMC Health Serv Res. 2016;16(a):383. Published 2016 Aug 15. doi:10.1186/s12913-016-1646-5
53. Neuman J, Korenstein D, Ross JS, et al. Prevalence of financial conflicts of interest among panel members producing clinical practice guidelines in Canada and United States: cross sectional study. Bmj. 2011 Oct 11;343. doi: https://doi.org/10.1136/bmj.d5621
54. Yang N, Yu Y, Zhang A, et al. Reporting, presentation and wording of recommendations in clinical practice guideline for gout: a systematic analysis. BMJ Open. 2019;9(1):e024315. Published 2019 Jan 29. doi:10.1136/bmjopen-2018-024315

For peer review only

55. Beauchemin M, Cohn E, Shelton RC. Implementation of Clinical Practice Guidelines in the Health Care Setting: A Concept Analysis. ANS Adv Nurs Sci. 2019;42(4):307-324. doi:10.1097/ANS.0000000000000263
56. Fretheim A, Schünemann HJ, Oxman AD. Improving the use of research evidence in guideline development: 3. Group composition and consultation process. Health Res Policy Syst. 2006;4:15. Published 2006 Nov 29. doi:10.1186/1478-4505-4-15
57. Kunz R, Fretheim A, Cluzeau F, et al. Guideline group composition and group processes: article 3 in Integrating and coordinating efforts in COPD guideline development. An official ATS/ERS workshop report. Proc Am Thorac Soc. 2012;9(5):229-233. doi:10.1513/pats.201208-056ST
58. Li SA, Yousefi-Nooraie R, Guyatt G, et al. A few panel members dominated guideline development meeting discussions: Social network analysis. J Clin Epidemiol. 2022;141:1-10. doi:10.1016/j.jclinepi.2021.09.023
59. Chen Y, Yang K, Marušic A, et al. A Reporting Tool for Practice Guidelines in Health Care: The RIGHT Statement. *Ann Intern Med*. 2017;166(2):128-132. doi:10.7326/M16-1565
60. Qaseem A, Forland F, Macbeth F, et al. Guidelines International Network: toward international standards for clinical practice guidelines. *Ann Intern Med*. 2012;156(7):525-531. doi:10.7326/0003-4819-156-7-201204030-00009
61. African GIN. Guidelines International Network African Community: Taking Leadership for Clinical Guidance for Africa. Available from: https://africaevidencenetwork.org/en/learning-space/article/138.
62. Mbuagbaw L, Zogo PO, Kredo T, et al. Cochrane Africa: a network of evidence-informed health-care decision making across sub-saharan Africa. *Pan Afr Med J*. 2018;29:196. Published 2018 Apr 3. doi:10.11604/pamj.2018.29.196.14521
63. Africa Cs. Cochrane south Africa. Available from: https://southafrica.cochrane.org/our-work/south-african-grade-network.
64. Africa Evidence Network. Available from: https://africaevidencenetwork.org/en/.
65. Joanna Briggs Institute. Available from: https://jbi.global/.
66. Pilla B, Jordan Z, Christian R, et al. JBI series paper 4: the role of collaborative evidence networks in promoting and supporting evidence-based health care globally: reflections from 25 years across 38 countries. *J Clin Epidemiol*. 2022;150:210-215. doi:10.1016/j.jclinepi.2022.04.009

For peer review only

67. Cochrane South Africa. South African GRADE Network. Available from: https://southafrica.cochrane.org/our-work/south-african-grade-network.

For peer review only

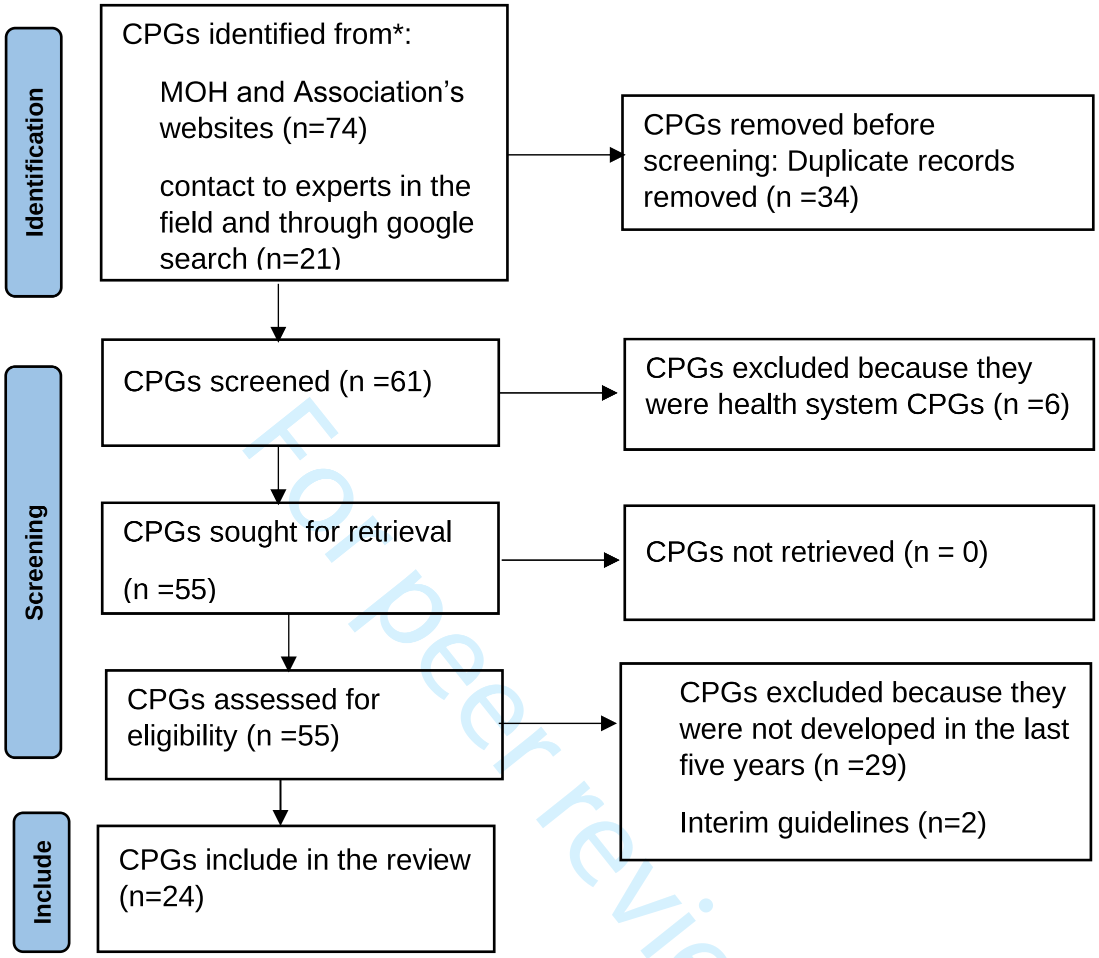

Figure 1: PRISMA-2020 flow diagram showing CPGs inclusion process

For peer review only

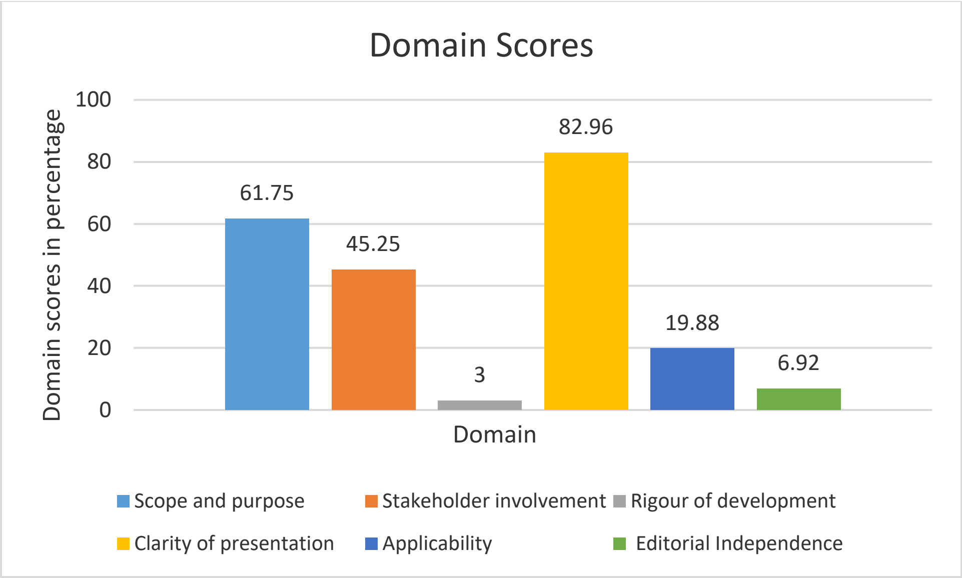

Figure 2: Mean domain scores of clinical practice guidelines

For peer review only

Supplementary file 1: AGREE II Instrument domains and items

Domain	Item no.	Item
1. Scope and purpose	1.	The guideline's overall objective(s) is (are) specifically described.
	2.	The health question(s) covered by the guideline is (are) specifically described.
	3.	The population (patients, public, etc.) the guideline is meant to apply is specifically described.
2. Stakeholder involvement	4.	The guideline development group includes individuals from all relevant professional groups.
	5.	The views and preferences of the target population (patients, the public, etc.) have been sought.
	6.	The target users of the guideline are clearly defined.
3. Rigour of development	7.	Systematic methods were used to search for evidence.
	8.	The criteria for selecting the evidence are clearly described.
	9.	The strengths and limitations of the body of evidence are clearly described.
	10.	The methods for formulating the recommendations are clearly described.
	11.	The health benefits, side effects, and risks have been considered in formulating the recommendations.
	12.	There is an explicit link between the recommendations and the supporting evidence.
	13.	Experts have externally reviewed the guideline before its publication.
	14.	A procedure for updating the guideline is provided.
4. Clarity of Presentation	15.	The recommendations are specific and unambiguous.
	16.	The different options for management of the condition or health issue are clearly presented.
	17.	Key recommendations are easily identifiable.
5. Applicability	18.	The guideline describes the facilitators and barriers to its application.
	19.	The guideline provides advice and/or tools on how the recommendations can be implemented.

For peer review only

	20.	The potential resource implications of applying the recommendations have been considered.
	21.	The guideline presents monitoring and/or auditing criteria.
6. Editorial Independence	22.	The views of the funding body have not influenced the guideline's content.
	23.	Competing interests of guideline development group members have been recorded and addressed
Overall guideline Assessment		Rate the overall quality of this guideline.
		I would recommend this guideline for use. (Yes, yes with modifications, no).

For peer review only

For peer review only

Supplementary file 2: Websites, databases and experts contacted in clinical practice guidelines search

	Disease	Databases	Contact persons/Experts
1.	HIV	Ministry of health<br>National aids control council (NCC)<br>Division of National AIDS and STI Control Programme (NASCOP)	Ministry of health officials<br>Respective heads
2.	T.B.	Ministry of health<br>Centre for Health Solutions (CHS) Kenya<br>Kenya Association for the Prevention of T.B. and Lung Diseases<br>Division of National T.B., Leprosy and Lung disease programme	Ministry of health officials respective heads
3.	Malaria	Ministry of health<br>The Division of National Malaria Programme (DNMP)	Ministry of health officials respective heads
4.	Covid-19	Ministry of health	Ministry of health officials
5.	Diarrheal diseases	Ministry of health	Ministry of health officials

For peer review only

6.	Infectious diseases	Infectious disease society of Kenya	Respective head
7.	Cardiovascular diseases	Ministry of health<br>Kenya cardiac association	Ministry of health officials<br>Respective disease head
8.	Cancer	Ministry of health<br>Kenya cancer association<br>National cancer institute of Kenya	Ministry of health officials<br>Respective head
9.	Diabetes mellitus	Ministry of health<br>Kenya diabetes association	Ministry of health officials
9.	Chronic respiratory diseases	Ministry of health	Ministry of health officials
10.	Cirrhosis	Ministry of health	Ministry of health officials
11.	Chronic kidney disease	Ministry of health<br>National kidney foundation<br>Kenya renal association	Ministry of health officials
12.	Nutritional disorders	Ministry of health	Ministry of health officials

For peer review only

13.	Maternal disorders	Ministry of health<br><br>Kenya obstetrical gynaecological society	Ministry of health officials
14.	Neonatal disorders	Ministry of health<br><br>Kenya paediatric association	Ministry of health officials
15.	Injuries	Ministry of health	Ministry of health officials

For peer review only

For peer review only

Supplementary file 3: Data extraction form

Items	Description of items	Date of extraction
Title		
Authors		
Year of publication		
Disease domain		
Scope		

For peer review only

Supplementary file 4: List of excluded clinical practice guidelines

DISEASE	Name of guideline	AUTHOR	YEAR	SCOPE	Reason for Exclusion
**COVID-19**	COVID-19 guidance on comprehensive HIV service delivery	NASCOP, C&T, MOH	2020	Prevention	Health system CPG
	Interim guidance on provision of services for non communicable diseases (NCDs) during the COVID-19 pandemic	MoH	2020	Management	Interim CPG
	Interim guidance for nutrition and dietetics management of covid-19 for health workers in treatment and isolation centres	MoH	2020	Management	Interim CPG
	Guidelines on continued provision of community health services in the context of corona virus	MoH	2020	Management	health system CPG
	Guidelines on use of antiretroviral drugs for treating and preventing HIV infection in Kenya	MoH & NASCOP	2016	Treatment	More than 5 years
**HIV**	A practical handbook for HIV managers and service providers on differentiated care	NASCOP	2016	Prevention	More than 5 years
	National guidelines for HIV testing and counselling in Kenya	MoH & NASCOP	2008	Diagnosis	More than 5 years
	The Kenya HIV testing services guidelines	MoH & NASCOP	2015	Diagnosis	More than 5 years
	National manual for the management of HIV-related opportunistic infections and conditions	MoH & NASCOP	2008	Integrated	More than 5 years
	National guidelines for PMTCT peer education and psychosocial support in Kenya	MoH	2012	prevention	More than 5 years

For peer review only

	Guidelines for prevention of mother to child transmission (PMTCT) of HIV/AIDS in Kenya	MoH	2012	prevention	More than 5 years
	National guidelines for HIV/STI programming with key populations	MoH	2014	Prevention	More than 5 years
**TB**	Guidelines for implementing TB-HIV collaborative activities in Kenya	MoH	2006	Integrated	More than 5 years
	Guidelines for the management of drug resistant tuberculosis in Kenya	MoH	2010	Management	More than 5 years
	National guidelines for the diagnosis, treatment and prevent of malaria in Kenya	MoH	2010	Integrated	More than 5 years
	National guidelines for the diagnosis, treatment and prevent of malaria in Kenya	MoH	2016	Integrated	More than 5 years
**Malaria**	Guidelines for malaria epidemic preparedness and response in Kenya	MoH	2020	Prevention	Health system CPG
**Cholera**	Policy guidelines for the management of diarrhoea in children below five years in Kenya	MoH	2014	Management	More than 5 years
	Guidelines on cholera control	MoH and WHO	2002	Prevention	More than 5 years
**DM**	National clinical guidelines for management of diabetes mellitus	MoH	2010	Integrated	More than 5 years
	Kenya national diabetes educators manual	MoH	2010	Prevention/management	More than 5 years
**Cancer**	National guidelines for establishment of cancer management centres in Kenya	MoH	2018	Prevention	Health systems
	National cancer specimen handling guidelines		2020	Prevention	not directly on patient care

For peer review only

	National guidelines for cancer management Kenya	MoH	2013	Integrated	More than 5 years
**KIDNEY D**	Guidelines for infection prevention in dialysis units	Kenya renal association		Prevention	More than 5 years
**KIDNEY D**	Policy guidelines on establishment and running of renal dialysis units	MoH	2015	Prevention	More than 5 years
**Maternal**	National guidelines for quality obstetrics and perinatal care	MoH	2016	Integrated	More than 5 years
**neonates/children**	National guidelines on essential newborn care	MoH	2016	Integrated	More than 5 years
	Integrated community case management for sick children under 5 years	MoH	2013	Integrated	More than 5 years
**Nutrition**	National maternal, infant and young child nutrition	MoH	2013	Integrated	More than 5 years
	National guideline for integrated management of acute malnutrition	MoH	2009	Integrated	More than 5 years
	Kenyan national guidelines on nutrition and HIV	MoH	2014	Management	More than 5 years
	Kenya national clinical nutrition and dietetics reference manual	MoH	2010	Integrated	More than 5 years
**injuries**	National guidelines on management of sexual violence in Kenya	MoH	2014	Integrated	More than 5 years
	Occupational safety and health policy guidelines for the health sector in Kenya	MoH	2014	Integrated	More than 5 years

For peer review only

**unclassified**	Clinical guidelines for management and referral of common conditions at level 2 and 3	MoH	2009	Integrated	More than 5 years
	Kenya essential medicines list	MoH	2019	1ntegrated	Health systems CPG

*TB-Tuberculosis

*MoH-Ministry of Health

*NASCOP-National AIDS and STIs Control Program

*HIV-Human Immunodeficiency Virus

*PMTCT- Prevention of Mother To Child Transmission

*WHO-World Health Organization

*STI-Sexually transmitted infection

*NCD-Non-Communicable disease

*COVID-19-Corona virus disease 2019

For peer review only

For peer review only

Supplementary file 5: Domain scores

	Domain	Mean percentage	SD	95% CI
**1.**	Scope and purpose	61.75	17.90	[54.19-69.31]
**2.**	Stakeholder involvement	45.25	12.41	[40.01-50.49]
**3.**	Rigour of development	3	5.66	[0.61-5.39]
**4.**	Clarity of presentation	82.96	10.92	[78.35-87.57]
**5.**	Applicability	19.88	15.53	[13.32-26.43]
**5.**	Editorial Independence	6.92	8.17	[3.47-10.37]

For peer review only

BMJ Open

Quality Assessment of Clinical Practice Guidelines in Kenya Using the AGREE II Tool:A Methodological Review

Journal:	*BMJ Open*
Manuscript ID	bmjopen-2023-074510.R1
Article Type:	Original research
Date Submitted by the Author:	20-Jun-2023
Complete List of Authors:	Sagam, Caleb; Kenya Medical Research Institute<br>Were, Lisa M.; Kenya Medical Research Institute<br>Otieno, Jenifer A.; Kenya Medical Research Institute<br>Mulaku, Mercy N.; Kenya Medical Research Institute; Stellenbosch University<br>Kariuki , Simon; Kenya Medical Research Institute<br>Ochodo, Eleanor; Kenya Medical Research Institute; Stellenbosch University
**Primary Subject Heading**:	Public health
Secondary Subject Heading:	Evidence based practice, Global health, Public health
Keywords:	Health Equity, Clinical Decision-Making, Health policy < HEALTH SERVICES ADMINISTRATION & MANAGEMENT, Health & safety < HEALTH SERVICES ADMINISTRATION & MANAGEMENT, Protocols & guidelines < HEALTH SERVICES ADMINISTRATION & MANAGEMENT

SCHOLARONE™
Manuscripts

I, the Submitting Author has the right to grant and does grant on behalf of all authors of the Work (as defined in the below author licence), an exclusive licence and/or a non-exclusive licence for contributions from authors who are: i) UK Crown employees; ii) where BMJ has agreed a CC-BY licence shall apply, and/or iii) in accordance with the terms applicable for US Federal Government officers or employees acting as part of their official duties; on a worldwide, perpetual, irrevocable, royalty-free basis to BMJ Publishing Group Ltd ("BMJ") its licensees and where the relevant Journal is co-owned by BMJ to the co-owners of the Journal, to publish the Work in this journal and any other BMJ products and to exploit all rights, as set out in our licence.

The Submitting Author accepts and understands that any supply made under these terms is made by BMJ to the Submitting Author unless you are acting as an employee on behalf of your employer or a postgraduate student of an affiliated institution which is paying any applicable article publishing charge ("APC") for Open Access articles. Where the Submitting Author wishes to make the Work available on an Open Access basis (and intends to pay the relevant APC), the terms of reuse of such Open Access shall be governed by a Creative Commons licence – details of these licences and which Creative Commons licence will apply to this Work are set out in our licence referred to above.

Other than as permitted in any relevant BMJ Author's Self Archiving Policies, I confirm this Work has not been accepted for publication elsewhere, is not being considered for publication elsewhere and does not duplicate material already published. I confirm all authors consent to publication of this Work and authorise the granting of this licence.

For peer review only

Quality Assessment of Clinical Practice Guidelines in Kenya Using the AGREE II Tool: A Methodological Review

*Caleb K. Sagam[1], Lisa M. Were[1], Jenifer A. Otieno[1], Mercy N. Mulaku[1,2,3], Simon Kariuki[1], Eleanor A. Ochodo[1,2]

Author affiliations:

[1]Centre for Global Health Research, Kenya Medical Research Institute, Kisumu, Kenya.

[2]Centre for Evidence-based Health Care, Division of Epidemiology and Biostatistics, Faculty of Medicine and Health Sciences, Stellenbosch University, Cape Town, South Africa.

[3]Department of Pharmacology, Clinical Pharmacy, and Pharmacy Practice, Faculty of Health Sciences, University of Nairobi, Kenya

*Caleb K. Sagam: sagamc7@gmail.com

Lisa M. Were: lisawere1997@gmail.com

Jenifer A. Otieno: jenipherakoth15@gmail.com

Mercy N. Mulaku: mercymulaku@gmail.com

Simon Kariuki: skariuki1578@gmail.com

Eleanor A. Ochodo: eleanor.ochodo@gmail.com

***Corresponding author**: Caleb K. Sagam sagamc7@gmail.com

: P.O. Box 1578-40100, KEMRI-CGHR, Kisumu, Kenya

Word count:3796 (excluding tables, references and cover page)

For peer review only

Abstract

Objective: To assess the quality of available and accessible national Clinical Practice Guidelines (CPGs) in Kenya using the Appraisal of Guidelines for Research and Evaluation II (AGREE II) tool.

Methods: We searched the websites of the Kenyan Ministry of Health, professional associations and contacted experts in relevant organisations. Our scope was guidelines on maternal, neonatal, nutritional disorders, injuries, communicable and non-communicable diseases in Kenya published in the last five years until 30th June 2022. Study selection and data extraction were done by three independent reviewers with disagreements resolved via discussion or with a senior reviewer We conducted a quality assessment using the online English version of AGREE II tool across six domains. Descriptive statistics were analysed using STATA software version 17. The primary outcome was the methodological quality of the included CPGs assessed by the AGREE II tool score.

Results: We retrieved 95 CPGs and included 24 in the analysis after screening for eligibility. The CPGs scored best in clarity of presentation and least in the rigour of development. In descending order, the appraisal scores (mean and CI) per domain were as follows: Clarity of presentation 82.96% (95% CI 78.35-87.57) with all guidelines scoring above 50%. Scope and purpose 61.75% (95% CI 54.19-69.31) with seven guidelines scoring less than 50%. Stakeholder involvement 45.25% (95% CI 40.01-50.49) with 16 CPGs scoring less than 50%. Applicability domain 19.88% (95% CI 13.32-26.43) with only one CPG scoring above 50%. Editorial independence 6.92% (95% CI 3.47-10.37) with no CPG scoring above 50% and Rigour of development 3% (95% CI 0.61-5.39) with no CPG scoring at least 50%.

Conclusion: Our findings suggest that the quality of CPGs in Kenya is limited mainly by the rigour of development, editorial independence, applicability and stakeholder involvement. Training initiatives on evidence-based methodology among guideline developers are needed to improve the overall quality of CPGs for better patient care.

Keywords: Clinical Practice Guidelines, Quality, AGREE II, Kenya, review.

For peer review only

Strengths and Limitations of this Study

- We used AGREE II instrument, an internationally used and validated tool for the quality of CPGs.
- Three independent reviewers rigorously appraised the CPGs and the final score was reached through a consensus.
- Poor reporting in identified CPGs limited our assessment with the AGREE II tool.
- Due to absence of a guideline clearing house in Kenya, searches were limited to available guideline repositories, websites and experts.

Protocol Preprint DOI: https://doi.org/10.31219/osf.io/3nu2q

Introduction

Clinical Practice Guidelines (CPGs) aid healthcare professionals in decision-making and provide a tool to implement recommended health policies at the point of care (1). According to the United States of America Institute of Medicine, CPGs are systematically developed recommendations that help healthcare practitioners make appropriate healthcare decisions to optimise patient care, informed by a systematic review of the evidence, assessment of benefits, harms and alternative care options (2). Good quality evidence-based guidelines developed or adapted robustly and transparently are essential in ensuring good quality healthcare (3).

Kenya, a low-middle income country with a life expectancy of about 66.7 years, is faced with health challenges, including a high burden of communicable diseases, an increasing burden of non-communicable diseases (NCDs), a high maternal & child mortality and injuries (4). Communicable diseases have remained the leading cause of morbidity and mortality (5). In 2020, mortality due to communicable diseases in the general population was 50.1%, a decline compared to 58.6% in 2016 (6). Mortality due to NCDs is on the rise, with 42.8% of deaths being attributed to NCDs in 2020, compared to 37% in 2016, while mortality attributed to injuries was 7.1% in 2020 (4). Four major NCDs, including cancer, diabetes, chronic respiratory and cardiovascular diseases, account for 33% of all the NCD deaths in Kenya, with other diseases such as mental health conditions on the increase (7). Mortality is mainly high among infants and young children and those above the age of

For peer review only

65 years, a trend similar in both males and females (6). According to High-Quality Technical Assistance for Results (HEART) organisation 2018 annual report, maternal and neonatal mortalities in Kenya account for 360/100,000 and 22/1000 live births, respectively (8).

A skilled and well-guided health workforce is needed to tackle the outlined disease burden in Kenya. The World Health Organization (WHO) defines core healthcare workers as medical doctors, nurses, and clinical officers/doctors assistants and recommends a minimum core health worker density threshold of 23 health workers per 10,000 population for effective service delivery (9, 10). As of 2020, Kenya's core health worker density was 16.6 health workers per 10,000, with 1.7 doctors per 10,000 population and 12.1 nurses per 10,000 population (6). With a shortage of health workers amidst a high disease burden, well-developed, implemented, and accessible CPGs are essential in guiding quality healthcare practices and improving the quality of life.

Previous studies have shown that CPGs improve health outcomes (11). In a study by Flarity *et al*, CPGs were found to reduce hospital length of stay (12). According to Ruseckaite *et al,* the use of CPGs in Australia led to an improvement in the nutritional status of children with cystic fibrosis (13). Implementation strategies of the CPGs have also been shown to improve health outcomes (3). An assessment of an international cohort of CPGs for glycaemic control in patients with type 2 diabetes mellitus highlighted the variation in the quality of CPGs and their effect on the quality of care (14). To our knowledge, this is the first evaluation of the methodological quality of CPGs in Kenya. We aimed to assess the methodological quality of available and accessible national CPGs in Kenya using the Appraisal of Guidelines for Research & Evaluation (AGREE II) tool (15); (Supplementary file 1). This review will inform the future development of high-quality evidence-based guidelines and recommend areas of strengthening the current CPGs in Kenya and beyond.

Methods

Eligibility criteria

Data on mortality and morbidity in Kenya are sparse and the available data are often not linked together to produce a comprehensive ranking of the disease burden. We, therefore, included available and accessible Kenyan CPGs on diseases or conditions under the broad health categories identified by the global burden of diseases, injuries, and risk

For peer review only

factors study 2019 (16). The broad health categories included communicable diseases, non-communicable diseases, maternal disorders, neonatal disorders, nutritional disorders, and injuries. We included the latest CPGs versions for screening, diagnosis, and treatment/management used in direct patient care for adults and children. We included CPGs published in English in the last five years until 30th June 2022. It is estimated that CPGs get outdated five years after their development (1).

We excluded guideline reports (manuscripts and any other published papers), summaries, press statements, or interim guidance documents because they are typically temporary and intended for provisional or emergency situations. Due to their urgent nature a rigorous evidence-based methodology may not be utilised hence they could have incomplete evidence or guidance with methods not reported fully (17). We also excluded health system guidelines because they are not used directly in patient care.

Identification of clinical guidelines

Kenya does not have a guideline clearing house to enable identification of all published and current health guidelines. CPGs are also rarely published in the scientific literature. We thus searched the websites of the Kenyan Ministry of Health, Health Associations and contacted relevant organisations' personnel for additional guidelines. We also searched the Kenya e-repository (for Kenyan government documents). We applied broad searches in Google, using the following search terms "clinical practice guidelines," "health guidelines," and "Kenya". We searched for guidelines in the websites of the ministry of health and various health associations by clicking the guidelines, standards or policy links. Guidelines were searched with the cut-off date of 30 July 2022. Only guidelines published within the last five years form this cut-off date were included. A detailed list of websites, databases, type of search and full search strategy used in Google can be found in supplementary file 2.

Patient and public involvement

The study design did not include patient and public involvement.

Screening and selection

For peer review only

We conducted CPG's screening and selection using an Excel spreadsheet, where we arranged all retrieved guidelines according to diseases and the latest guideline for each disease was picked for appraisal. C.S, J.O and L.W screened the title and publication year of CPG documents for eligibility. We retrieved CPGs that meet the eligibility criteria for quality assessment. We resolved discrepancies on inclusion through consensus or discussion with E.O. Selection process details are provided in the Preferred Reporting Items for Systematic Reviews and Meta-Analysis 2020 (PRISMA) flow chart below (18). The protocol for this review was registered in Open Science Framework (OSF) (19).

Data extraction

C.S, J.O and L.W independently extracted data using a Google form into an Excel worksheet (supplementary file 3). We extracted the general information of the included guidelines, including title, year of publication, author, disease, and scope of the guideline. We resolved discrepancies through consensus or discussion with E.O.

Quality assessment

C.S, J.O and L.W independently assessed the quality of CPGs using the AGREE II tool, a validated and widely used tool, recommended by WHO for assessing the quality, transparency and rigour of CPGs (15). An international group of guideline developers and researchers first developed AGREE II in 2003. It was published in 2009, revised in 2017, and is currently used globally in appraising CPGs (20). It contains six domains and 23 items (15). The six domains evaluated include scope and purpose, stakeholder involvement, the rigour of development, (evidence-based methodology), clarity of presentation, applicability and editorial independence (conflict of interest).

The reviewers conducted the appraisal and scored each item against a seven-point Likert scale on the online version of AGREE II. We scored one if none of the criteria for an item was met or the item was reported very poorly. We scored seven when the item met all the criteria and was well-reported.

The primary outcome of this study was the methodological quality of the CPGs assessed by AGREE II score. The quality scores were determined by calculating the scaled domain percentages for each CPG as outlined in the AGREE II tool (15). We have reported each item's average appraiser score and overall assessment scores. We also calculated the

For peer review only

average scores for the 23 items and the overall assessment score for each CPG using STATA software version 17 (Stata Corp LLC, College TX). We present our results in tables as means, confidence intervals, standard deviations and graphs. The overall assessment score is not an average of individual domain scores but an independent domain.

Results

Search results

We retrieved 95 CPGs from the Ministry of Health Kenya, different divisions and health associations' websites. After the removal of duplicates, we identified 61 guidelines. During full-text screening, we excluded 37 guidelines that did not meet the inclusion criteria. We finally included 24 CPGs (figure 1). A list of excluded CPGs can be found in supplementary file 4.

General characteristics of included guidelines

The description of the general characteristics of the assessed guidelines is illustrated in (Table 1). Most of the guidelines were integrated (these are guidelines containing more than one purpose i.e., Diagnosis and screening and treatment or management) (n=19 CPGs). Six CPGs were published in 2017, six in 2018, two in 2019, five in 2020, four in 2021, and one in 2022. The Ministry of Health Kenya authored majority of the guidelines.

For peer review only

Table 1: Characteristics of included clinical practice guidelines

Disease domain	Title of the guideline	Author	Year of publication	Scope
COVID-19	Guidelines on case management of COVID-19 in Kenya (21)	MoH	2021	Integrated
	Guidelines to be used by occupational therapists in rehabilitation of patients during COVID-19 pandemic (19)	MoH	2020	Management
HIV	Guidelines on use of antiretroviral drugs for treating and preventing HIV infection in Kenya (22)	MoH & NASCOP	2018	Integrated
	National guidance on tetanus prevention in voluntary medical male circumcision settings in Kenya (23)	MoH	2017	Prevention
TB	National guidelines on management of tuberculosis in children (24)	MoH	2017	Integrated
	Integrated guideline for tuberculosis, leprosy and lung disease (25)	MoH	2021	Integrated
	Field guide on systematic screening of active TB in Kenya (26)	MoH	2017	Screening
Malaria	Guidelines for the diagnosis, treatment & prevention of malaria in Kenya (27)	MoH	2020	Integrated
CVD	Kenya national guidelines for cardiovascular diseases management (28)	MoH	2018	Integrated
Eye	National guidelines for the management of glaucoma (29)	MoH	2020	Integrated
	Guidelines for the screening and management of retinopathy of prematurity in Kenya (30)	MoH	2018	Integrated
Diabetes	National clinical guidelines for management of diabetes mellitus (31)	MoH	2018	Integrated
	Guidelines for screening and management of diabetic retinopathy (32)	MoH	2017	Integrated

For peer review only

Cancer	Kenya national cancer treatment protocols (33)	MoH	2019	Integrated
	Retinoblastoma best practice guidelines (34)	MoH	2019	Integrated
	National cancer screening guidelines (35)	MoH	2018	Screening
Renal disease	Guidelines for the management of emergencies in dialysis (36)	Kenya renal association	2017	Management
Mental health	National tele-mental health guidelines (37)	MoH	2021	Integrated
Respiratory diseases	Kenya asthma management guideline (38)	MoH	2021	Integrated
Maternal health	Guidelines for postnatal care (39)	MoH	2017	Integrated
Paediatrics	Guidelines on the management of paediatric patients during COVID-19 pandemic (40)	MoH	2020	Integrated
	Basic paediatric protocols(41)	MoH	2022	Integrated
Sickle Cell	National guidelines for control and management of sickle cell disease in Kenya (42)	MoH	2020	Integrated
Nutrition	National guidelines for healthy diets and physical activity (43)	MoH	2017	Prevention

*MoH-Ministry of Health

*NASCOP-National AIDS and STIs Control Program

*HIV-Human Immunodeficiency Virus

*TB-Tuberculosis

*CVD-cardiovascular diseases

For peer review only

Findings on AGREE II guideline quality score per domain

In reference to Table 2, Figure 2 and Supplementary file 5, we present the findings from the highest-scoring domain to the least-scoring domain:

Clarity of presentation

The mean score was 82.96% (95% CI 78.35-87.57) with all guidelines scoring above 50%. The overall mean of this domain was the highest among all domains. Recommendations were specific and unambiguous. Key recommendations were easily identifiable and different options for managing the health condition were stated in varying ways. Items 15 and 16 scored above six and 17 scored above five (Table 3).

Scope and purpose

The mean score in this domain was 61.75% (95% CI 54.19-69.31). Most guidelines scored well in this domain with only seven scoring less than 50%. Under domain one items one, two and three scored above four out of the possible seven. This is because the health questions, the guideline objective and the population to whom the guideline was applied were described in most guidelines. However, adequate information was not reported on the expected benefit or outcome and health care setting. The description of health intents was not well written and clear in some guidelines.

Stakeholder involvement

The mean score in this domain was 45.25% (95% CI 40.01-50.49). This domain score was lower than domain one because most guidelines did not involve evidence-based experts and patients in the development process. Sixteen CPGs scored less than 50%. In domain two, items four and six scored above four out of possible seven (Table 3). Item five had a score of one, this is because the views and preferences of the target population were only sought in 2 out of the 24 CPGs. Disciplines and a description of the member's role in the guideline development group were missing in most guidelines.

Applicability

The applicability domain mean score was 19.88% (95% CI 13.32-26.43) with only one CPG scoring above 50%. Most of the CPGs did not describe the facilitators and barriers of its application or provide advice on how tools can be put into practice. They also did not

For peer review only

present monitoring and evaluation criteria and potential resource implications of the recommendations. Under domain five, all the items scored below three out of the possible seven, especially in items 18 and 20 (Table 3).

Editorial independence

The mean score was 6.92% (95% CI 3.47-10.37) with no CPG scoring above 50%. The scores in this domain were the second lowest after the rigour of development. Items in this domain scored less than two, this is because information on funding was either not stated or not clear. Conflict of interest was poorly reported, this contributed to the low score in domain six because information on the declaration of competing interest by the guideline development group was not provided. Though a third of the guidelines mentioned the funding bodies, the role of the funding bodies in the guideline development process was not provided.

Rigour of development

This domain has the highest number of items and plays a key role in developing CPGs. The mean was 3% (95% CI 0.61-5.39) with no CPG scoring at least 50%. The overall domain score was poor with the highest score of 26%. A total of eight CPGs scored zero percent in this domain which points out serious shortcomings.

Under domain three, all the items from items seven to 14 scored less than two out of the possible seven (Table 3). Systematic methods were not used to search for evidence, the criteria for selecting the evidence were not described and the strengths and limitations of the evidence were not described. In addition, methods for formulating recommendations were not clear, there was no information on whether health benefits, side effects, harms and risks were considered in formulating recommendations. An explicit link between recommendation and supporting evidence was also not presented and there was no information to show that the guideline was externally reviewed before its publication. All these factors led to a score of less than two for each item.

In this study, the scores of the 24 CPGs on the six domains, the only two domains scored above 50% is scope and purpose and clarity of presentation. These guidelines scored low in editorial independence, applicability and rigour of development.

For peer review only

Table 2: Domain scores for each CPG

CPG Name	Domain percentages scores						Average (%)
	1	2	3	4	5	6	
Guidelines for screening and management of diabetic retinopathy (32)	96	72	26	81	42	28	67
National guidelines for the management of glaucoma (29)	98	63	11	96	29	0	61
Integrated guideline for tuberculosis, leprosy and lung disease (25)	67	56	1	93	42	8	56
Guidelines for the screening and management of retinopathy of prematurity in Kenya (30)	81	63	5	91	31	8	56
National guidelines for healthy diets and physical activity (43)	81	44	0	94	53	3	56
Field guide on systematic screening of active TB in Kenya (26)	56	30	1	89	49	0	50
National cancer screening guidelines (35)	70	54	2	94	7	11	50
Kenya asthma management guideline (38)	78	52	1	87	38	3	50
National guidelines for control and management of sickle cell disease in Kenya (42)	70	54	1	85	13	0	50
National guidance on tetanus prevention in voluntary medical male circumcision settings in Kenya (23)	44	30	8	72	29	17	44
National guidelines on management of tuberculosis in children (24)	69	52	0	76	19	0	44
Guidelines for the diagnosis, treatment & prevention of malaria in Kenya (27)	70	44	1	91	14	14	44
Kenya national cancer treatment protocols (33)	44	50	0	94	11	6	44
Retinoblastoma best practice guidelines (34)	54	41	4	85	8	3	44
Guidelines on the management of pediatric patients during COVID-19* pandemic (40)	57	28	6	80	0	0	44
National clinical guidelines for management of diabetes mellitus (31)	63	46	0	83	11	25	44
Basic pediatric protocols (41)	50	41	1	78	18	6	44

For peer review only

Guidelines to be used by occupational therapists in rehabilitation of patients during covid-19* pandemic (19)	65	52	2	80	7	0	39
Kenya national guidelines for cardiovascular diseases management (28)	37	37	0	93	14	6	39
Guidelines for postnatal care (39)	65	48	0	78	17	3	39
Guidelines on case management of COVID-19* in Kenya (21)	56	30	1	81	4	0	33
Guidelines on the use of antiretroviral drugs for treating and preventing HIV infection in Kenya (22)	48	44	0	80	7	19	33
National tele-mental health guidelines (37)	35	31	0	54	14	6	33
Guidelines for management of emergencies in dialysis (36)	28	24	1	56	0	0	28

*TB-Tuberculosis

*HIV-Human Immunodeficiency Virus

*COVID-19-Corona virus disease 2019

For peer review only

Table 3: Quality of CPGs per item of the AGREE II tool

Variable	Mean	Std. dev.	[95% conf. interval]
Item 1	5.03	1.43	[4.42 5.63]
Item 2	4.06	1.57	[3.39 4.72]
Item 3	4.54	1.25	[4.01 5.07]
Item 4	4.26	1.35	[3.69 4.84]
Item 5	1.32	0.68	[1.03 1.61]
Item 6	5.66	1.49	[4.93 6.18]
Item 7	1.13	0.35	[0.98 1.27]
Item 8	1.07	0.22	[0.98 1.16]
Item 9	1.01	0.07	[0.99 1.04]
Item 10	1.11	0.29	[0.99 1.23]
Item 11	1.08	0.28	[0.96 1.20]
Item 12	1.24	0.71	[0.94 1.54]
Item 13	1.14	0.37	[0.98 1.29]
Item 14	1.68	1.35	[1.11 2.25]
Item 15	6.10	0.79	[5.76 6.43]
Item 16	6.32	0.75	[6.00 6.63]
Item 17	5.61	0.95	[5.21 6.01]
Item 18	1.61	0.93	[1.22 2.00]
Item 19	2.94	0.98	[2.53 3.36]
Item 20	1.49	0.85	[1.13 1.85]
Item 21	2.71	1.95	[1.89 3.53]
Item 22	1.82	0.98	[1.40 2.23]
Item 23	1.00	0.00	[1.00 1.00]
Overall assessment	3.74	0.56	[3.50 3.97]

For peer review only

DISCUSSION

We assessed the quality of 24 CPGs in Kenya published in the last five years untill 30th June 2022 using the AGREE II tool across six domains: clarity of presentation, scope and purpose, stakeholder involvement, applicability, editorial independence and rigour of development. The CPGs scored best in clarity of presentation (82.96% (95% CI 78.35-87.57)) and scored least in the rigour of development (3% (95% CI 0.61-5.39)).

Similar to our results, a study done in China reported challenges in the rigour of development (44). The quality of CPGs is heavily affected by the use of evidence-based methods (45). In a systematic review of the effect of evidence-based CPGs on the quality of care, evidence-based clinical guidelines improved the quality of care (3). It is recommended that guideline recommendations be made based on the best available evidence using an evidence-to-decision framework (EtD) (46). EtD frameworks (such as the Grading of Recommendations, Assessments and Evaluations (GRADE EtD) and WHO INTEGRATE (EtD) provide a systematic and explicit process linking the best available evidence to formulating the recommendations (47-49).

According to a study on publishing CPGs, external review plays a critical role in enhancing scientific validity, clarity and feasibility of the guidelines before formal publication (50). External review is a critical step in enhancing accountability during guideline development. It provides an opportunity for additional inputs and a critical review of the guideline by a specialist before its publication. Our findings show that over two-thirds of the guidelines did not report on the procedure and frequency of updating the guideline. The body of knowledge is constantly changing, therefore there is a need to review CPGs. Updating CPGs is very important as it enhances the validity of the recommendations (51). According to a systematic review of guidance for updating CPGs two to three years is the recommended time frame between publishing a guideline and commencing the updating process (52).

Similar to our results, a study done in China found shortcomings in editorial independence (44). This can be attributed to conflict-of-interest disclosure policies and whether they have been developed or challenges that come with implementation. Transparency is a key aspect in the development of CPGs (50). According to a study on reporting financial

For peer review only

conflicts of interest in CPGs, conflict of interest is a threat because adequate literature is documented on the influence of uncontrolled conflict of interest on recommendations (53). The influence of external activities such as grant funding and ownership of commercial entities have created a bias and affected the process of making recommendations (54). Efforts to balance guideline development groups (GDGs) have improved however included experts in the industry involved in commercial activities which makes it difficult to rule out a conflict of interest (50). Reporting is also an integral part of ensuring transparency, because when a conflict of interest was done but not reported it brings confusion and it will always be assumed that it was not done which may not be the reality (55).

Poor applicability threatens the usability of the guidelines at the facility level. The applicability domain goes beyond the methodological quality and covers resource implication, which is key to successfully implementing each guideline (56). Similar to our study on appraisal of clinical guidelines for recurrent urinary tract infections also showed low scores in this domain (52). Monitoring and evaluation plan ensures the guidelines serve their intended purpose. In many cases the guideline developers may not be responsible for the direct implementation of the guideline therefore it is important to stipulate how the monitoring and auditing will be done to ensure adequate implementation of the guideline.

GDG composition is one of the most important aspects of guideline development (49). A GDG necessitates the expertise of healthcare professionals, patient input on their needs and preferences and methodologists and librarians skilled in gathering, summarising, and interpreting evidence (57). Structured education in this methodological area will be required, given the increasing demands and expertise required to manage complex guideline development projects (58). The focus of the guidelines should influence the number of group members and the balance of disciplines. When deciding on the group's composition, all potential stakeholders should be identified, including healthcare professionals who are directly involved in the clinical management of patients in various healthcare settings (for example, primary and secondary care), policymakers who may need to make resource utilisation decisions and patients (58). The decision must then be made about which categories of participants to include in the guideline group. Guidelines

For peer review only

developers must frequently balance the desire for broad representation with the need for a cohesive working group. Small groups may lack experience among their members, while larger groups may lack cohesiveness and be difficult to lead (59).

Limitations of the study

Our study only included available guidelines published in the last five years on the broad health categories; communicable diseases, non-communicable diseases, maternal disorders, neonatal disorders, nutritional disorders and injuries. This may have excluded other key guidelines. There is also no clear way of differentiating appraisal scores in cases where there was poor reporting and no reporting, this is because both are rated 1 according to AGREE II. The tool also relies on the subjective judgments of the assessors, which may vary depending on their expertise and expectations.

Accessibility of some guidelines was also a challenge due to lack of a common repository for CPGs or guideline clearing house in Kenya. Furthermore, Kenyan guidelines are not indexed in the electronic databases that are commonly used, such as PubMed or Cochrane Library, therefore we limited our search to Google, websites and contacting experts. With the limited sources to retrieve CPGs we did not involve an information specialist but employed broad searches. With this our search may have been limited but we believe we identified a representative sample to judge the quality of existing CPGs.

Implication for practice

Given our findings, we recommend that awareness of the Reporting Items for Practice Guidelines in HealThcare (RIGHT) statement among guideline developers be improved (60). This could be done through conferences, webinars and training workshops by evidence-based groups in Africa (such as Guidelines International Network(61, 62), Cochrane Africa (63, 64), Africa Evidence Network (65), Joanna Briggs Institute (66, 67) and GRADE networks in Africa (68)). Increased awareness will enable better and more transparent reporting of CPGs. More training initiatives among GDG members on aspects of AGREE II and RIGHT guidelines and the involvement of a guideline methodologist will improve the rigour of development, reporting and overall methodological quality of CPGs in Kenya.

For peer review only

Implications for research

A qualitative study to explore guideline developers' knowledge and perspectives on guideline development methods would be useful in knowing the barriers and enablers to guideline development in Kenya. Qualitative research among the users of the guidelines (healthcare workers) would also shed light on barriers to the impact of the guidelines on health practices and outcomes. Impact evaluations on the effect of low- or high-quality CPGs on healthcare in Kenya and other LMIC settings are also needed.

Conclusion

The quality of CPGs in Kenya is limited mainly by the rigour of development, editorial independence, applicability and stakeholder involvement. Training initiatives on evidence-based methodology among guideline developers are needed to improve the overall quality of CPGs for better patient care.

Ethics approval

We did not seek formal ethical approval since this is a methodological review based on publicly available CPGs.

Author Contributions

Conceptualisation of the study: E.O.

Writing of the first draft: C.S., J.O., and L.W.

Critical review of the manuscript J.O., L.W., M.M., S.K, and E.O.

Writing the final draft of the manuscript CS.

Approval of the final draft of the manuscript: C.S., L.W., J.O., M.M., S.K., E.O.

Funding sources/ sponsors

This study is funded under the UK MRC African Research Leaders award number MR/T008768/1.

The funding organizations had no role in the development of this study.

Conflict of interest

None of the authors declared a conflict of interest.

Data availability statement

For peer review only

Data are available upon reasonable request.

References

1. Gary S. Gronseth, David Gloss, Julie Cox, et al. American Academy of Neurology Clinical Practice Guideline Process Manual.: American Academy of Neurology; 2017.
2. Graham R MM, Miller Wolman D, Greenfield S, et al. Clinical Practice Guidelines We Can Trust. Institute of Medicine (US) Committee on Standards for Developing Trustworthy Clinical Practice Guidelines, Washington (DC): National Academies Press (US); 2011.
3. Lugtenberg M, Burgers JS, Westert GP. Effects of evidence-based clinical practice guidelines on quality of care: a systematic review. Qual Saf Health Care. 2009;18(5):385-392. doi:10.1136/qshc.2008.028043
4. MoH. Kenya Health Sector Strategic Plan, June 2018-2023 Transforming Health Systems: Achieving Universal Health Coverage by 2022. Nairobi: Ministry of Health, Kenya,2018. Available from: https://www.health.go.ke/wp-content/uploads/2020/11/Kenya-Health-Sector-Strategic-Plan-2018-231.pdf, accessed 20th April 2022.
5. Bigna JJ, Noubiap JJ. The rising burden of non-communicable diseases in sub-Saharan Africa. Lancet Glob Health. 2019;7(10): e1295-e1296. doi:10.1016/S2214-109X (19)30370-5
6. MoH. Kenya Health Sector strategic plan 2018-2023, mid-term review synthesis report. Monitoring Progress Towards Attainment of Universal Health Coverage. Nairobi: Ministry of Health, Kenya, 2021. Available from: https://www.health.go.ke/wp-content/uploads/2022/01/KHSSP-MTR-Syntheis-Report2021.pdf, accessed 20th April 2022.
7. MoH. Kenya National Strategy for the Prevention and Control of Non-Communicable Diseases 2015 - 2020. Nairobi: Ministry of Health, Kenya, 2015. Available from: https://www.worlddiabetesfoundation.org/sites/default/files/Kenya%20national%20strategy%20for%20NCDs%202015-2020%20%281%29.pdf, accessed 20th April 2022.
8. Results H-QTAf. Reducing Maternal and Neonatal Deaths in Kenya. Annual Review – post-April 2018.; 2018. Available from: https://www.heart-resources.org/wp-content/uploads/2018/12/MNH_Programme_2018_Annual_Review.pdf, accessed 30th April 2022.

For peer review only

9. WHO. Monitoring the building blocks of health systems. A Handbook of Indicators and their measurement strategies 2010. Geneva: World Health Organization. Available from:https://apps.who.int/iris/bitstream/handle/10665/258734/9789241564052-eng.pdf, accessed 20th April 2022.
10. WHO. Global strategy on human resources for health workforce 2030: Geneva: World Health Organization 2016. Available from: https://apps.who.int/iris/bitstream/handle/10665/250368/9789241511131-eng.pdf. accessed 20th April 2022.
11. Grimshaw JM, Russell IT. Effect of clinical guidelines on medical practice: a systematic review of rigorous evaluations. Lancet. 1993;342(8883):1317-1322. doi:10.1016/0140-6736(93)92244-n
12. Flarity K, Rhodes WC, Berson AJ, et al. Guideline-Driven Care Improves Outcomes in Patients with Traumatic Rib Fractures. Am Surg. 2017;83(9):1012-1017. PMID: 28958283
13. Ruseckaite R, Pekin N, King S, et al. Evaluating the impact of 2006 Australasian Clinical Practice Guidelines for nutrition in children with cystic fibrosis in Australia. Respir Med. 2018;142:7-14. doi:10.1016/j.rmed.2018.07.007
14. Holmer HK, Ogden LA, Burda BU, et al. Quality of clinical practice guidelines for glycemic control in type 2 diabetes mellitus. PLoS One. 2013;8(4):e58625. doi:10.1371/journal.pone.0058625
15. Brouwers MC, Kho ME, Browman GP, et al. AGREE II: advancing guideline development, reporting and evaluation in health care. CMAJ. 2010;182(18):E839-E842. doi:10.1503/cmaj.090449
16. GBD 2019 Diseases and Injuries Collaborators. Global burden of 369 diseases and injuries in 204 countries and territories, 1990-2019: a systematic analysis for the Global Burden of Disease Study 2019. Lancet. 2020;396(10258):1204-22. PMID: 33069326 PMCID: PMC7567026 DOI: 10.1016/S0140-6736(20)30925-9
17. Dagens A, Louise Sigfrid, Erhui Cai, et al. Scope, quality, and inclusivity of clinical guidelines produced early in the covid-19 pandemic: rapid review. BMJ. 2020;10.1136(1936).
18. Page MJ, McKenzie JE, Bossuyt PM, et al. The PRISMA 2020 statement: an updated guideline for reporting systematic reviews. BMJ. 2021;372: n71. Published 2021 Mar 29. PMID: 33782057 PMCID: PMC8005924 DOI: 10.1136/bmj. n71

For peer review only

19. Sagam CK, Were L, Otieno JA, et al. Quality Assessment of Clinical Practice Guidelines in Kenya Using the AGREE II Tool: Methodological Review Protocol https://osf.io/preprints/3nu2q/.DOI: 10.31219/osf.io/3nu2q
20. Brouwers MC, Kho ME, Browman GP, et al. AGREE-II-Users-Manual-and-23-item-Instrument-2009-Update. 2017. Available at: https://www.agreetrust.org/wp-content/uploads/2017/12/AGREE-II-Users-Manual-and-23-item-Instrument-2009-Update-2017.pdf
21. MoH. Guidelines on Case Management of COVID-19 in Kenya. Nairobi: Ministry of Health, Kenya, 2021. Available at: https://www.health.go.ke/wp-content/uploads/2021/10/Final-guidelines-on-the-Management-of-Covid-19-in-Kenya-2021-Edition.pdf, accessed 20th April 2022.
22. MoH. Guidelines on Use of Antiretroviral Drugs for Treating and Preventing HIV Infection in Kenya. Nairobi: Ministry of Health, Kenya, 2018. Available from: https://www.nascop.or.ke/new-guidelines/, accessed 20th April 2022.
23. MoH. National guidance on tetanus prevention in voluntary medical male circumcision settings in Kenya. Nairobi: Ministry of Health, Kenya, 2017. Available from: https://www.nascop.or.ke/new-guidelines/, accessed 20th April 2022.
24. MoH. National Guidelines on Management of Tuberculosis in Children. Nairobi: Ministry of Health, Kenya, 2017. Available from: https://www.nltp.co.ke/guideline/, accessed 20th April 2022.
25. MoH. Integrated Guideline for Tuberculosis, Leprosy and Lung Disease. Nairobi: Ministry of Health, Kenya, 2021. Available from: https://www.nltp.co.ke/guideline/, accessed 20th April 2022.
26. MoH. Field guide on systematic screening of active TB in Kenya. Nairobi: Ministry of Health, Kenya, 2017. Available from: https://chskenya.org/wp-content/uploads/2017/09/Active-Case-Finding-Booklet-1.pdf, accessed 20th April 2022.
27. MoH. Guidelines for the diagnosis, treatment and prevention of malaria in Kenya. Nairobi: Ministry of Health, Kenya, 2020.
28. MoH. Kenya National Guidelines for Cardiovascular Diseases Management. Nairobi: Ministry of Health, Kenya 2018. Available from: http://guidelines.health.go.ke:8000/media/Kenya_National_Guidelines_for_Cardiovascular_Diseases_Management.pdf, accessed 20th April 2022.

For peer review only

29. MoH. National guidelines for the management of Glaucoma. Nairobi: Ministry of Health, Kenya, 2020. Available from: https://www.health.go.ke/wp-content/uploads/2020/12/National-Guidelines-for-Management-of-Glaucoma-Nov.2020.pdf, accessed 20th April 2022.
30. MoH. National guidelines for screening and management of retinopathy of prematurity. Nairobi: Ministry of Health, Kenya, 2018. Available from: https://www.health.go.ke/wp-content/uploads/2021/02/National-Guidelines-for-Screening-and-Management-of-Retinopathy-of-Prematurity.2018.pdf, accessed 20th April 2022.
31. MoH. Kenya National Clinical Guidelines for Management of Diabetes Mellitus. Nairobi: Ministry of Health, Kenya, 2018.
32. MoH. Guidelines for Screening and Management of Diabetic Retinopathy. Nairobi: Ministry of Health, Kenya, 2017. Available from: https://www.health.go.ke/wp-content/uploads/2017/11/Guidelines-for-Screening-and-Management-of-Diabetic-Retinopathy-in-Kenya.pdf, accessed 20th April 2022.
33. MoH. Kenya National Cancer Treatment Protocols. Nairobi: Ministry of Health, Kenya, 2019. Available from: https://www.health.go.ke/wp-content/uploads/2019/09/National-treatment-Protocols-2019.pdf, accessed 20th April 2022
34. MoH. Retinoblastoma-Best-Practice-Guidelines. Nairobi: Ministry of Health, Kenya, 2019. Available from: https://www.health.go.ke/wp-content/uploads/2021/02/RETINOBLASTOMA-Best-Practice-Guidelines-2019.pdf, accessed 20th April 2022.
35. MoH. Kenya National Cancer Screening Guidelines. Nairobi: Ministry of Health, Kenya, 2018. Available from: https://www.health.go.ke/wp-content/uploads/2019/02/National-Cancer-Screening-Guidelines-2018.pdf, accessed 20th April 2022.
36. MoH. Guidelines for the management of emergencies in dialysis. Nairobi: Ministry of Health, Kenya, 2017. Available from: accessed 20th April 2022.
37. MoH. National Tele Mental-Health Guidelines. Nairobi: Ministry of Health, Kenya, 2021. Available from: https://www.health.go.ke/wp-content/uploads/2023/02/MOH-TELE-MENTAL-HEALTH-GUIDELINESUpdated-1.pdf, accessed 20th April 2022.
38. MoH. Kenya Asthma Management Guideline. Nairobi: Ministry of Health, Kenya, 2021. Available from: https://www.nltp.co.ke/guideline/, accessed 20th April 2022.

For peer review only

39. MoH. Guidelines for Postnatal Care. Nairobi: Ministry of Health, Kenya, 2017. Available from: https://familyhealth.go.ke/wp-content/uploads/2018/02/Guidelines-for-postnatal-care-to-mothers-and-newborns-2016-18-12-2016B.pdf,accessed 20th April 2022.
40. MoH. Guidelines for Management of Pediatric Patients During COVID-19 Pandemic. Nairobi: Ministry of Health, Kenya, 2020. Available from: https://www.health.go.ke/wp-content/uploads/2020/06/PAEDIATRIC-Covid-Guidelines-Final.pdf, accessed 20th April 2022.
41. MoH. Basic pediatric protocol 5th edition. Nairobi: Ministry of Health, Kenya, 2022. Available from: https://www.coursehero.com/file/134237800/Basic-Paediatric-Protocols-JAN-27-2022-DRAFT-SWpdf/ accessed 20th April 2022.
42. MoH. National Guidelines for the Management of Sickle Cell Disease in Kenya. Nairobi: Ministry of Health, Kenya, 2020. Available from: https://ncdak.org/wp-content/uploads/2021/07/national-guidelines-for-control-and-management-of-sickle-cell-disease-in-Kenya-.pdf, accessed 20th April 2022.
43. MoH. National Guidelines for Healthy Diets and Physical Activity. Nairobi: Ministry of Health, Kenya, 2017. Available from: http://www.nutritionhealth.or.ke/wp-content/uploads/Downloads/National%20Guidelines%20for%20Healthy%20Diets%20and%20Physical%20Activity%202017.pdf, accessed 20th April 2022.
44. Gao Y, Wang J, Luo X, et al. Quality appraisal of clinical practice guidelines for diabetes mellitus published in China between 2007 and 2017 using the AGREE II instrument. BMJ Open. 2019;9(9):e022392. Published 2019 Sep 4. doi:10.1136/bmjopen-2018-022392
45. Graham ID, Harrison MB. Evaluation and adaptation of clinical practice guidelines. Evid Based Nurs. 2005;8(3):68-72. doi:10.1136/ebn.8.3.68
46. Li SA, Yousefi-Nooraie R, Guyatt G, et al. A few panel members dominated guideline development meeting discussions: Social network analysis. *J Clin Epidemiol*. 2022;141:1-10. doi:10.1016/j.jclinepi.2021.09.023
47. Alonso-Coello P, Schünemann HJ, Moberg J, et al. GRADE Evidence to Decision (EtD) frameworks: a systematic and transparent approach to making well informed healthcare choices. 1: Introduction. BMJ. 2016;353:i2016. Published 2016 Jun 28. doi:10.1136/bmj.i2016
48. Rehfuess EA, Stratil JM, Scheel IB, et al. The WHO-INTEGRATE evidence to decision framework version 1.0: integrating WHO norms and values and a

For peer review only

complexity perspective. *BMJ Glob Health*. 2019;4(Suppl 1):e000844. Published 2019 Jan 25. doi:10.1136/bmjgh-2018-000844

49. Atkins D, Best D, Briss PA, et al. Grading quality of evidence and strength of recommendations. BMJ. 2004;328(7454):1490. doi:10.1136/bmj.328.7454.1490
50. Yang N, Zhao W, Qi W, et al. Publishing clinical prActice GuidelinEs (PAGE): Recommendations from editors and reviewers. J Evid Based Med. 2022;15(2):136-141. doi:10.1111/jebm.12479
51. Smith CA, Toupin-April K, Jutai JW, et al. A Systematic Critical Appraisal of Clinical Practice Guidelines in Juvenile Idiopathic Arthritis Using the Appraisal of Guidelines for Research and Evaluation II (AGREE II) Instrument. PLoS One. 2015;10(9):e0137180. Published 2015 Sep 10. doi:10.1371/journal.pone.0137180
52. Pat JJ, Witte LPW, Steffens MG, et al. Quality appraisal of clinical guidelines for recurrent urinary tract infections using AGREE II: a systematic review. Int Urogynecol J. 2022;33(5):1059-1070. doi:10.1007/s00192-022-05089-6
53. Shnier A, Lexchin J, Romero M, et al. Reporting of financial conflicts of interest in clinical practice guidelines: a case study analysis of guidelines from the Canadian Medical Association Infobase. BMC Health Serv Res. 2016;16(a):383. Published 2016 Aug 15. doi:10.1186/s12913-016-1646-5
54. Neuman J, Korenstein D, Ross JS, et al. Prevalence of financial conflicts of interest among panel members producing clinical practice guidelines in Canada and United States: cross sectional study. Bmj. 2011 Oct 11;343. doi: https://doi.org/10.1136/bmj.d5621
55. Yang N, Yu Y, Zhang A, et al. Reporting, presentation and wording of recommendations in clinical practice guideline for gout: a systematic analysis. BMJ Open. 2019;9(1):e024315. Published 2019 Jan 29. doi:10.1136/bmjopen-2018-024315
56. Beauchemin M, Cohn E, Shelton RC. Implementation of Clinical Practice Guidelines in the Health Care Setting: A Concept Analysis. ANS Adv Nurs Sci. 2019;42(4):307-324. doi:10.1097/ANS.0000000000000263
57. Fretheim A, Schünemann HJ, Oxman AD. Improving the use of research evidence in guideline development: 3. Group composition and consultation process. Health Res Policy Syst. 2006;4:15. Published 2006 Nov 29. doi:10.1186/1478-4505-4-15
58. Kunz R, Fretheim A, Cluzeau F, et al. Guideline group composition and group processes: article 3 in Integrating and coordinating efforts in COPD guideline

For peer review only

development. An official ATS/ERS workshop report. Proc Am Thorac Soc. 2012;9(5):229-233. doi:10.1513/pats.201208-056ST

59. Li SA, Yousefi-Nooraie R, Guyatt G, et al. A few panel members dominated guideline development meeting discussions: Social network analysis. J Clin Epidemiol. 2022;141:1-10. doi:10.1016/j.jclinepi.2021.09.023
60. Chen Y, Yang K, Marušic A, et al. A Reporting Tool for Practice Guidelines in Health Care: The RIGHT Statement. *Ann Intern Med*. 2017;166(2):128-132. doi:10.7326/M16-1565
61. Qaseem A, Forland F, Macbeth F, et al. Guidelines International Network: toward international standards for clinical practice guidelines. *Ann Intern Med*. 2012;156(7):525-531. doi:10.7326/0003-4819-156-7-201204030-00009
62. African GIN. Guidelines International Network African Community: Taking Leadership for Clinical Guidance for Africa. Available from: https://africaevidencenetwork.org/en/learning-space/article/138.
63. Mbuagbaw L, Zogo PO, Kredo T, et al. Cochrane Africa: a network of evidence-informed health-care decision making across sub-saharan Africa. *Pan Afr Med J*. 2018;29:196. Published 2018 Apr 3. doi:10.11604/pamj.2018.29.196.14521
64. Africa Cs. Cochrane south Africa. Available from: https://southafrica.cochrane.org/our-work/south-african-grade-network.
65. Africa Evidence Network. Available from: https://africaevidencenetwork.org/en/.
66. Joanna Briggs Institute. Available from: https://jbi.global/.
67. Pilla B, Jordan Z, Christian R, et al. JBI series paper 4: the role of collaborative evidence networks in promoting and supporting evidence-based health care globally: reflections from 25 years across 38 countries. *J Clin Epidemiol*. 2022;150:210-215. doi:10.1016/j.jclinepi.2022.04.009
68. Cochrane South Africa. South African GRADE Network. Available from: https://southafrica.cochrane.org/our-work/south-african-grade-network.

Figure 1: PRISMA-2020 flow diagram showing CPGs inclusion process

Figure 2: Mean domain scores of clinical practice guidelines

For peer review only

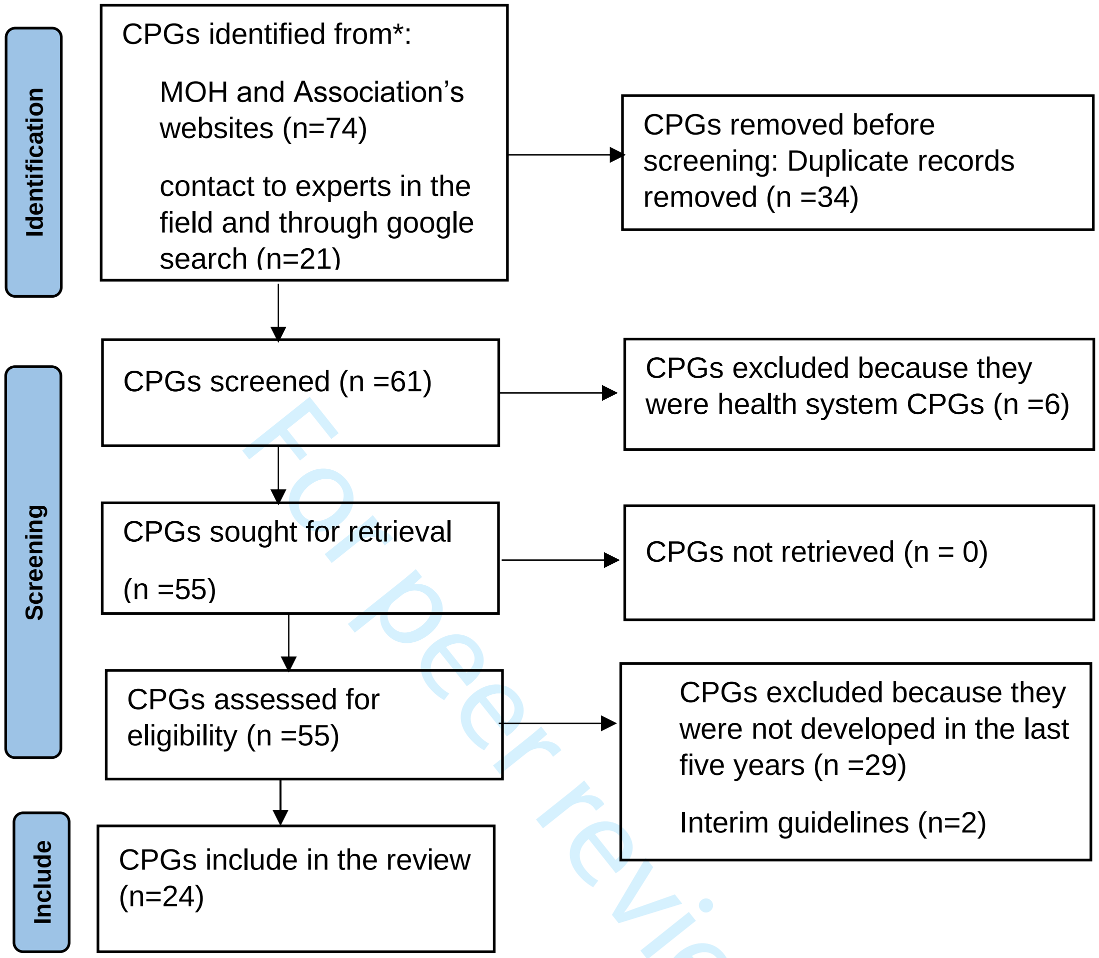

Figure 1: PRISMA-2020 flow diagram showing CPGs inclusion process

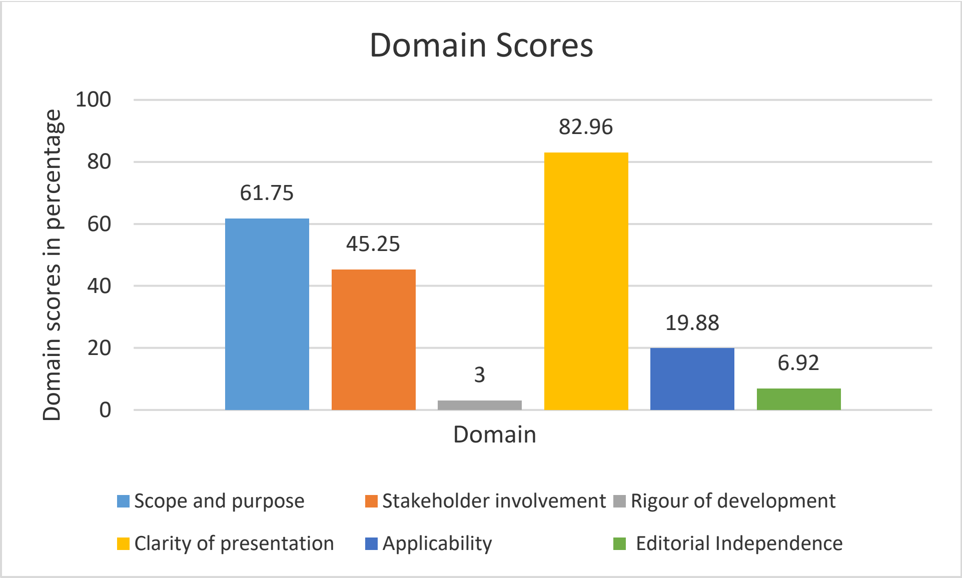

Figure 2: Mean domain scores of clinical practice guidelines

peer review only

Supplementary file 1: AGREE II Instrument domains and items

Domain	Item no.	Item
1. Scope and purpose	1.	The guideline's overall objective(s) is (are) specifically described.
	2.	The health question(s) covered by the guideline is (are) specifically described.
	3.	The population (patients, public, etc.) the guideline is meant to apply is specifically described.
2. Stakeholder involvement	4.	The guideline development group includes individuals from all relevant professional groups.
	5.	The views and preferences of the target population (patients, the public, etc.) have been sought.
	6.	The target users of the guideline are clearly defined.
3. Rigour of development	7.	Systematic methods were used to search for evidence.
	8.	The criteria for selecting the evidence are clearly described.
	9.	The strengths and limitations of the body of evidence are clearly described.
	10.	The methods for formulating the recommendations are clearly described.
	11.	The health benefits, side effects, and risks have been considered in formulating the recommendations.
	12.	There is an explicit link between the recommendations and the supporting evidence.
	13.	Experts have externally reviewed the guideline before its publication.
	14.	A procedure for updating the guideline is provided.
4. Clarity of Presentation	15.	The recommendations are specific and unambiguous.
	16.	The different options for management of the condition or health issue are clearly presented.
	17.	Key recommendations are easily identifiable.
5. Applicability	18.	The guideline describes the facilitators and barriers to its application.
	19.	The guideline provides advice and/or tools on how the recommendations can be implemented.

For peer review only

	20.	The potential resource implications of applying the recommendations have been considered.
	21.	The guideline presents monitoring and/or auditing criteria.
6. Editorial Independence	22.	The views of the funding body have not influenced the guideline's content.
	23.	Competing interests of guideline development group members have been recorded and addressed
Overall guideline Assessment		Rate the overall quality of this guideline.
		I would recommend this guideline for use. (Yes, yes with modifications, no).

For peer review only

For peer review only

Supplementary file 2: Websites, databases and experts contacted in clinical practice guidelines search

	Disease	Databases	Contact persons/Experts	Type of search
1.	HIV	Ministry of health<br>National aids control council (NCC)<br>Division of National AIDS and STI Control Programme (NASCOP)	Ministry of health officials<br>Respective heads	Hand searching
2.	T.B.	Ministry of health<br>Centre for Health Solutions (CHS) Kenya<br>Kenya Association for the Prevention of T.B. and Lung Diseases<br>Division of National T.B., Leprosy and Lung disease programme	Ministry of health officials respective heads	Hand searching
3.	Malaria	Ministry of health<br>The Division of National Malaria Programme (DNMP)	Ministry of health officials respective heads	Hand searching

For peer review only

4.	Covid-19	Ministry of health	Ministry of health officials	Hand searching
5.	Diarrheal diseases	Ministry of health	Ministry of health officials	Hand searching
6.	Infectious diseases	Infectious disease society of Kenya	Ministry of health officials<br>Respective head	Hand searching
7.	Cardiovascular diseases	Ministry of health<br>Kenya cardiac association	Ministry of health officials<br>Respective disease head	Hand searching
8.	Cancer	Ministry of health<br>Kenya cancer association<br>National cancer institute of Kenya	Ministry of health officials<br>Respective head	Hand searching

For peer review only

9.	Diabetes mellitus	Ministry of health<br>Kenya diabetes association	Ministry of health officials	Hand searching
9.	Chronic respiratory diseases	Ministry of health	Ministry of health officials	Hand searching
10.	Cirrhosis	Ministry of health	Ministry of health officials	Hand searching
11.	Chronic kidney disease	Ministry of health<br>National kidney foundation<br>Kenya renal association	Ministry of health officials	Hand searching
12.	Nutritional disorders	Ministry of health	Ministry of health officials	Hand searching
13.	Maternal disorders	Ministry of health<br>Kenya obstetrical gynaecological society	Ministry of health officials	Hand searching
14.	Neonatal disorders	Ministry of health<br>Kenya paediatric association	Ministry of health officials	Hand searching

For peer review only

15.	Injuries	Ministry of health	Ministry of health officials	Hand searching
16		Google		Search strategy used-See below

For peer review only

The following search strategy was used to search for CPGs in Google

1.	Clinical practice guidelines
2.	Health guidelines
3.	Clinical guidelines
4.	Guidelines
5.	Clinical protocols
6.	Treatment protocols
7.	Management protocols
8.	1 OR 2 OR 3 AND 26
9.	1 OR 2 OR 3 OR 4 OR 5 OR 6 AND 26
10.	1 OR 2 OR 3 OR 4 OR 5 OR 6 AND 26 AND HIV/AIDS
11.	1 OR 2 OR 3 OR 4 OR 5 OR 6 AND 26 AND Tuberculosis
12.	1 OR 2 OR 3 OR 4 OR 5 OR 6 AND 26 AND Malaria
13.	1 OR 2 OR 3 OR 4 OR 5 OR 6 AND 26 AND Covid-19
14.	1 OR 2 OR 3 OR 4 OR 5 OR 6 AND 26 AND Diarrheal diseases
15.	1 OR 2 OR 3 OR 4 OR 5 OR 6 AND 26 AND Infectious diseases
16.	1 OR 2 OR 3 OR 4 OR 5 OR 6 AND 26 AND Cardiovascular diseases

For peer review only

17.	1 OR 2 OR 3 OR 4 OR 5 OR 6 AND 26 AND Cancer
18.	1 OR 2 OR 3 OR 4 OR 5 OR 6 AND 26 AND Diabetes mellitus
19.	1 OR 2 OR 3 OR 4 OR 5 OR 6 AND 26 AND Chronic respiratory diseases
20.	1 OR 2 OR 3 OR 4 OR 5 OR 6 AND 26 AND Cirrhosis
21.	1 OR 2 OR 3 OR 4 OR 5 OR 6 AND 26 AND Chronic kidney disease
22.	1 OR 2 OR 3 OR 4 OR 5 OR 6 AND 26 AND Nutritional disorders
23.	1 OR 2 OR 3 OR 4 OR 5 OR 6 AND 26 AND Maternal disorders
24.	1 OR 2 OR 3 OR 4 OR 5 OR 6 AND 26 AND Neonatal disorders
25.	1 OR 2 OR 3 OR 4 OR 5 OR 6 AND 26 AND Injuries
26.	Kenya

For peer review only

Supplementary file 3: Data extraction form

Items	Description of items	Date of extraction
**Title**		
**Authors**		
**Year of publication**		
**Disease domain**		
**Scope**		

For peer review only

Supplementary file 4: List of excluded clinical practice guidelines

DISEASE	Name of guideline	AUTHOR	YEAR	SCOPE	Reason for Exclusion
**COVID-19**	COVID-19 guidance on comprehensive HIV service delivery	NASCOP, C&T, MOH	2020	Prevention	Health system CPG
	Interim guidance on provision of services for non communicable diseases (NCDs) during the COVID-19 pandemic	MoH	2020	Management	Interim CPG
	Interim guidance for nutrition and dietetics management of covid-19 for health workers in treatment and isolation centres	MoH	2020	Management	Interim CPG
	Guidelines on continued provision of community health services in the context of corona virus	MoH	2020	Management	health system CPG
	Guidelines on use of antiretroviral drugs for treating and preventing HIV infection in Kenya	MoH & NASCOP	2016	Treatment	More than 5 years
**HIV**	A practical handbook for HIV managers and service providers on differentiated care	NASCOP	2016	Prevention	More than 5 years
	National guidelines for HIV testing and counselling in Kenya	MoH & NASCOP	2008	Diagnosis	More than 5 years
	The Kenya HIV testing services guidelines	MoH & NASCOP	2015	Diagnosis	More than 5 years
	National manual for the management of HIV-related opportunistic infections and conditions	MoH & NASCOP	2008	Integrated	More than 5 years
	National guidelines for PMTCT peer education and psychosocial support in Kenya	MoH	2012	prevention	More than 5 years

For peer review only

	Guidelines for prevention of mother to child transmission (PMTCT) of HIV/AIDS in Kenya	MoH	2012	prevention	More than 5 years
	National guidelines for HIV/STI programming with key populations	MoH	2014	Prevention	More than 5 years
**TB**	Guidelines for implementing TB-HIV collaborative activities in Kenya	MoH	2006	Integrated	More than 5 years
	Guidelines for the management of drug resistant tuberculosis in Kenya	MoH	2010	Management	More than 5 years
	National guidelines for the diagnosis, treatment and prevent of malaria in Kenya	MoH	2010	Integrated	More than 5 years
	National guidelines for the diagnosis, treatment and prevent of malaria in Kenya	MoH	2016	Integrated	More than 5 years
**Malaria**	Guidelines for malaria epidemic preparedness and response in Kenya	MoH	2020	Prevention	Health system CPG
**Cholera**	Policy guidelines for the management of diarrhoea in children below five years in Kenya	MoH	2014	Management	More than 5 years
	Guidelines on cholera control	MoH and WHO	2002	Prevention	More than 5 years
**DM**	National clinical guidelines for management of diabetes mellitus	MoH	2010	Integrated	More than 5 years
	Kenya national diabetes educators manual	MoH	2010	Prevention/management	More than 5 years
**Cancer**	National guidelines for establishment of cancer management centres in Kenya	MoH	2018	Prevention	Health systems
	National cancer specimen handling guidelines		2020	Prevention	not directly on patient care

For peer review only

	National guidelines for cancer management Kenya	MoH	2013	Integrated	More than 5 years
**KIDNEY D**	Guidelines for infection prevention in dialysis units	Kenya renal association		Prevention	More than 5 years
**KIDNEY D**	Policy guidelines on establishment and running of renal dialysis units	MoH	2015	Prevention	More than 5 years
**Maternal**	National guidelines for quality obstetrics and perinatal care	MoH	2016	Integrated	More than 5 years
**neonates/children**	National guidelines on essential newborn care	MoH	2016	Integrated	More than 5 years
	Integrated community case management for sick children under 5 years	MoH	2013	Integrated	More than 5 years
**Nutrition**	National maternal, infant and young child nutrition	MoH	2013	Integrated	More than 5 years
	National guideline for integrated management of acute malnutrition	MoH	2009	Integrated	More than 5 years
	Kenyan national guidelines on nutrition and HIV	MoH	2014	Management	More than 5 years
	Kenya national clinical nutrition and dietetics reference manual	MoH	2010	Integrated	More than 5 years
**injuries**	National guidelines on management of sexual violence in Kenya	MoH	2014	Integrated	More than 5 years
	Occupational safety and health policy guidelines for the health sector in Kenya	MoH	2014	Integrated	More than 5 years

For peer review only

**unclassified**	Clinical guidelines for management and referral of common conditions at level 2 and 3	MoH	2009	Integrated	More than 5 years
	Kenya essential medicines list	MoH	2019	1ntegrated	Health systems CPG

*TB-Tuberculosis

*MoH-Ministry of Health

*NASCOP-National AIDS and STIs Control Program

*HIV-Human Immunodeficiency Virus

*PMTCT- Prevention of Mother To Child Transmission

*WHO-World Health Organization

*STI-Sexually transmitted infection

*NCD-Non-Communicable disease

*COVID-19-Corona virus disease 2019

For peer review only

For peer review only

Supplementary file 5: Domain scores

	Domain	Mean percentage	SD	95% CI
**1.**	Scope and purpose	61.75	17.90	[54.19-69.31]
**2.**	Stakeholder involvement	45.25	12.41	[40.01-50.49]
**3.**	Rigour of development	3	5.66	[0.61-5.39]
**4.**	Clarity of presentation	82.96	10.92	[78.35-87.57]
**5.**	Applicability	19.88	15.53	[13.32-26.43]
**5.**	Editorial Independence	6.92	8.17	[3.47-10.37]

For peer review only
